# Vesicle-mediated transport-related genes predict the prognosis and immune microenvironment in hepatocellular carcinoma

**DOI:** 10.7150/jca.94902

**Published:** 2024-05-13

**Authors:** Zhiyue Ye, Yang Wang, Ruixin Yuan, Ran Ding, Yaxin Hou, Luomeng Qian, Sihe Zhang

**Affiliations:** 1 Department of Cell Biology, School of Medicine, Nankai University, Tianjin, 300071, China.; 2 School of Biomedical Sciences and Engineering, South China University of Technology, Guangzhou International Campus, Guangzhou 511442, China.

**Keywords:** Vesicle-mediated transport-related genes, Prognostic signature, Immune microenvironment, Drug sensitivity, Hepatocellular carcinoma

## Abstract

**Background**: Liver hepatocellular carcinoma (LIHC) is one of the leading causes of cancer-related death. The prognostic outcomes of advanced LIHC patients are poor. Hence, reliable prognostic biomarkers for LIHC are urgently needed.

**Methods**: Data for vesicle-mediated transport-related genes (VMTRGs) were profiled from 338 LIHC and 50 normal tissue samples downloaded from The Cancer Genome Atlas (TCGA). Univariate Cox regression and Least Absolute Shrinkage and Selection Operator (LASSO) regression analyses were performed to construct and optimize the prognostic risk model. Five GEO datasets were used to validate the risk model. The roles of the differentially expressed genes (DEGs) were investigated via Kyoto Encyclopedia of Genes and Genomes (KEGG) and Gene Ontology (GO) enrichment analyses. Differences in immune cell infiltration between the high- and low-risk groups were evaluated using five algorithms. The “pRRophetic” was used to calculate the anticancer drug sensitivity of the two groups. Transwell and wound healing assays were performed to assess the role of GDP dissociation inhibitor 2 (GDI2) on LIHC cells.

**Results**: A total of 166 prognosis-associated VMTRGs were identified, and VMTRGs-based risk model was constructed for the prognosis of LIHC patients. Four VMTRGs (GDI2, DYNC1LI1, KIF2C, and RAB32) constitute the principal components of the risk model associated with the clinical outcomes of LIHC. Tumor stage and risk score were extracted as the main prognostic indicators for LIHC patients. The VMTRGs-based risk model was significantly associated with immune responses and high expression of immune checkpoint molecules. High-risk patients were less sensitive to most chemotherapeutic drugs but benefited from immunotherapies. *In vitro* cellular assays revealed that GDI2 significantly promoted the growth and migration of LIHC cells.

**Conclusions**: A VMTRGs-based risk model was constructed to predict the prognosis of LIHC patients effectively. This risk model was closely associated with the immune infiltration microenvironment. The four key VMTRGs are powerful prognostic biomarkers and therapeutic targets for LIHC.

## Introduction

Liver hepatocellular carcinoma (LIHC) is a major histological type of liver malignancy, and has become a major cause of cancer-related death 1. Although current surgery and drug therapies have improved the survival of LIHC patients at an early stage, the prognostic outcomes of advanced LIHC patients are still unsatisfactory 2, 3. Early detection and precise therapy are urgently needed based on deeply understanding the tumor biology and immunity of LIHC.

Vesicle-mediated transport is a fundamental process for maintaining membrane-enclosed organelle homeostasis in eukaryotic cells. It is essential to control proper cellular signaling, nutrient uptake and waste disposal through three main steps: vesicle formation, vesicle trafficking, and vesicle fusion 4, 5. Growing evidences have indicated that alterations in vesicle-mediated transport-related genes (VMTRGs) not only cause various organelle defects and cellular dysfunctions but also implicated in the development and progression of cancer cells 6-9. For example, gene mutations in the RAB GTPase family are associated with colorectal cancer development and progression 10, 11. RAB37 is one of the key drivers of vesicle trafficking. Its overexpression facilitates the secretion of chitinase 3-like 1 (CHI3L1) in immune cells and induces the activation of M2 macrophages, leading to an abominable protumor microenvironment 12. The trafficking of CD147 membrane antigen, mediated by deregulated RAB GTPase activation, is believed to influence the cell adhesion and junction stability 13, 14. Moreover, recent studies have indicated that dysregulated expression of certain VMTRGs causes uncontrolled growth, invasion, and metastasis of cancer cells 15-17. VMTRGs-mediated macropinocytosis also plays a crucial role in the delivery of therapeutic genes for ovarian cancer therapy 18. Although the VMTRGs contribute to malignancy, the impact of VMTRGs on LIHC prognosis has not been determined. Therefore, determining the prognostic potential of VMTRGs for LIHC is highly important.

Evidences show that the tumor microenvironment is modulated by exosomes, leading to the influence in therapeutic response and clinical outcome 19, 20. Immune checkpoint inhibitors (ICIs) have variable efficacy in the treatment of LIHC. Emerging evidence suggest that vesicle trafficking may play a crucial role in immune regulation. For example, the thyroid adenoma-associated gene (THADA) is required for the residency of PD-L1 in the Golgi, and this coat protein complex II (COPII)-associated mechanism maintains PD-L1 highly expression in tumor cells. THADA mediate the interaction of PD-L1, a cargo protein, with the specific cargo adaptor protein SEC24A, a module involved in COPII-mediated vesicle trafficking. Targeting THADA substantially enhanced the T-cell-mediated cytotoxicity and increased CD8^+^ T-cell infiltration in mouse tumor tissues 21. Therefore, whether other VMTRGs act on the immune microenvironment of LIHC should be investigated.

In this study, we established a VMTRGs signature for the prognostic prediction of LIHC and evaluated its association with the immune microenvironment. The VMTRGs-based risk model has high potential for predicting the outcomes and treatment response of LIHC patients. We revealed that four hub genes (GDI2, DYNC1LI1, KIF2C, and RAB32) in this risk model are potential prognostic biomarkers and therapeutic targets, and the expression of GDP dissociation inhibitor 2 (GDI2) promoted the malignant phenotypes of LIHC cells.

## Materials and Methods

### Study population and data preprocessing

Clinical information and mRNA sequencing data (FPKM values) from 338 LIHC and 50 normal tissue samples (Table S1) were downloaded from The Cancer Genome Atlas (TCGA) (https://portal.gdc.cancer.gov/) database. The expression profile and prognostic data of patients in the validation cohort (Microarray GPL13158: GSE116174; GPL3921/GPL571: GSE14520) were downloaded from the Gene Expression Omnibus (GEO) (https://www.ncbi.nlm.nih.gov/gds) database. A total of 722 VMTRGs were extracted from the Reactome gene sets (https://reactome.org): REACTOME_VESICLE_MEDIATED_TRANSPORT (Table S2). For the TCGA cohort, we transformed the FPKM values into TPM values for in-depth analysis.

### Establishment of consistent clustering based on VMTRGs

To identify prognosis-related genes, univariate Cox regression analysis was performed by using the “coxPH” function of “survival” in R. In total, 166 candidate prognostic genes were screened from the 722 VMTRGs genes (Table S3).

### Construction and validation of a VMTRGs-based signature

Based on the TCGA database, 166 prognostic candidate VMTRGs were screened via Least Absolute Shrinkage and Selection Operator (LASSO) Cox regression analysis to reduce redundancy and avoid model overfitting 22. To predict the OS of LIHC patients, target genes were ultimately selected to construct a prognostic risk model. The risk score formula was as follows:



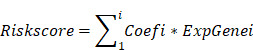



For the TCGA dataset, LIHC patients were split into high- and low-risk groups based on the median risk score from the cohort sample. The feasibility of the prognostic risk model was validated by using the aforementioned dataset from the GEO database. K‒M analysis was used to assess the difference in survival outcome between the high- and low-risk groups. To assess the independence of VMTRGs-based signature, univariate and multivariate Cox regression analyses were performed by using SPSS software. Sangerbox (http://www.sangerbox.com/tool) was used to analyze the risk score and other clinical data, including gender, age, and tumor stage.

### Construction and evaluation of the nomogram

By using “rms” in R, we performed a multivariate analysis of the DEGs that were distinctly associated with OS in TCGA-LIHC patients. Calibration plots were generated to evaluate the accuracy of the nomogram for predicting survival in LIHC patients.

### Immune infiltration analysis and assessment of drug sensitivity

The ESTIMATE algorithm “estimate” in R was used to calculate the immune score, stromal score, estimate score and fraction of tumor purity. The CIBERSORT, xCELL, EPIC, TIMER, QUANTISEQ and MCPCOUNTER algorithms in TIMER 2.0 (https://cistrome.shinyapps.io/timer/) were used to analyze the correlation between the VMTRGs-based risk score and immune cell infiltration level. Single-sample Gene Set Enrichment Analysis (ssGSEA) was also conducted by using the “GSVA” package. An inhibitory concentration of 50% was determined as the IC50. The IC50s of the drugs were calculated by using “pRRophetic” in R for LIHC patients from the TCGA dataset.

### Functional enrichment analysis

The “DESeq2” package in R was used to analyze the DEGs between the high- and low-risk groups. Gene Set Enrichment Analysis (GSEA) was performed by using GSEA v4.0 at significance thresholds of FDR<0.25 and *p*<0.05. The “clusterProfiler” package in R was used for Gene Ontology (GO) and Kyoto Encyclopedia of Genes and Genomes (KEGG) enrichment analyses of the DEGs.

### Cell lines

The human LIHC cell lines MHCC-97H and HepG2, obtained from the Type Culture Collection of the Chinese Academy of Sciences (China), were routinely cultured (37°C, DMEM with fetal bovine serum, 10% v/v) in normoxic (5% CO2) incubators (Thermo).

### RNA interference

The siRNA pool targeting GDI2 gene and NC‐siRNA (scrambled siRNA) were synthesized by GenePharma Co., Ltd. The siRNA sequences were shown in **Table 1**. LIHC cells were transiently transfected with the indicated siRNA pool for 12 hours by using Lipofectamine® 2000 (11668027, Life Technologies) according to the manufacturer's protocol, after which the cells were cultured for 48 hours before analysis. For each siRNA transfection, a 50 nM (final concentration) siRNA pool was used, and the knockdown efficiency was assessed via Western blotting.

### Quantitative real-time PCR

Total RNA was isolated using Trizol reagent (Takara Bio, Japan) from cultured cells. mRNA (1.0 μg) was used for cDNA synthesis by reverse transcription system (TransGen Biotech, China). Real-time polymearse chain reaction was performed using Hieff^TM^ qPCR SYBR^®^ Green Master Mix (YEASEN Biotechnology, Shanghai, China) in a Roche LightCycler 96 detection system. The mRNA expression of VMTRGs genes (GDI2, DYNC1LI1, KIF2C, and RAB32) was detected; the expression of GAPDH was used as the loading control. 2^-ΔΔCt^ method was used to determine relative fold changes for the expression of mRNAs. The primer sequences were shown in **Table 1**. Assays were performed in triplicate.

### Western blot

Cultured LIHC cells were washed with PBS and then lysed in RIPA lysis buffer (10 mM Tris-HCl, pH 7.4; 150 mM NaCl; 1 mM EDTA; 0.1% SDS; and 1% Triton X-100). After sonication (2 s each cycle) three times, the cell debris was removed via centrifugation at 10,000 rpm for 10 min. The protein concentration in the supernatant was measured by using a BCA assay (23227, Thermo). The protein samples were resolved via SDS‒PAGE, blotted onto PVDF membranes and subsequently incubated with primary Abs (anti-GDI2 Ab, 1:2000; 60078-1-lg, Proteintech; anti-Tubulin Ab, 1:5000; R23623, Zenbio) overnight at 4 °C. After incubating with the corresponding secondary Abs, the target proteins were visualized by enhanced chemiluminescence (ECL) detection reagents.

### CCK-8 assay

Cell proliferation was evaluated by using a CCK-8 kit (BMU106, SuperKine) according to the manufacturer's instructions. LIHC cells with or without siRNA transfection were reseeded in 96-well plates (2×10^3^ cells/well) and cultured for 24 h at 37°C. After the addition of CCK-8 solution (10 μL/well) and incubation for 2 hours, the absorbance (450 nm) in each well was measured by a microplate reader (FLUOstar Omega; BMG Labtech).

### Scratch-migration assay

LIHC cells with/without siRNA transfection were grown to a monolayer in 12-well plates (0.6% gelatin coated) and starved overnight with DMEM containing 0.1% FBS. After the cell monolayer was scraped with a sterile micropipette tip, complete medium (10% FBS) was added (t=0). After 24 hours of incubation, the cells were photographed under a phase-contrast microscope, and the wounded area was measured and calculated by using ImageJ software.

### Matrigel-coated transwell invasion assay

LIHC cells with or without siRNA transfection were harvested by trypsinization and reseeded (1×10^5^) in serum-free medium in Matrigel-coated transwell inserts (8 μm pore size, BK Falcon). The lower chamber was filled with 10% FBS medium. After 24 h of incubation, the cells on the filter in the upper chamber were removed with a cotton swab, and the cells on the underside were stained with a crystal violet solution and counted under a microscope. The number of invading cells was calculated by ImageJ.

### Statistical analysis

All R package analyses were executed by using R Studio software (V4.3.1). We used 'ggplot2' in R to construct the volcano plot. Correlations were determined by Pearson correlation analysis. The log-rank test was used to compare the K‒M survival curves. ROC analysis was performed by using Sangerbox. Statistical significance was established when ****p<0.0001, ***p<0.001, **p<0.01, *p<0.05, and -, *p*>=0.05. All the experiments were repeated three times.

## Results

### The VMTRGs-based prognostic model for LIHC

**Figure 1:**
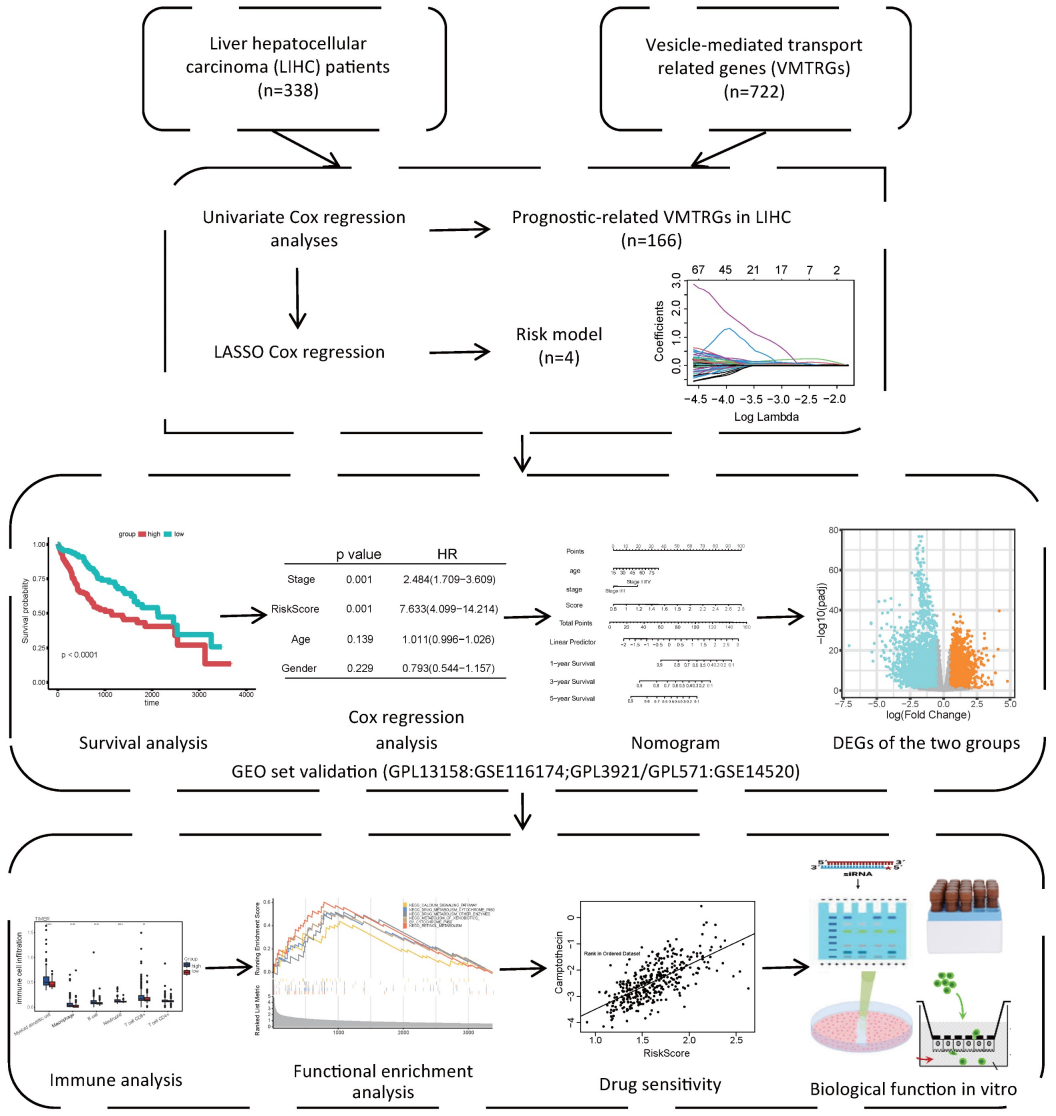
Study flowchart. The gene expression and clinical information of 338 LIHC patients were first obtained from the TCGA database. A total of 166 candidate prognosis-related genes were screened from the 722 VMTRGs by univariate Cox regression analysis. A signature based on the 4 VMTRGs was subsequently established via LASSO Cox regression analysis. To assess the prognostic value of this signature, the survival of LIHC patients was analyzed, and a nomogram was constructed. Finally, the VMTRGs-based risk score model was further assessed by immune analysis, functional enrichment and drug sensitivity analysis.

**Figure 2:**
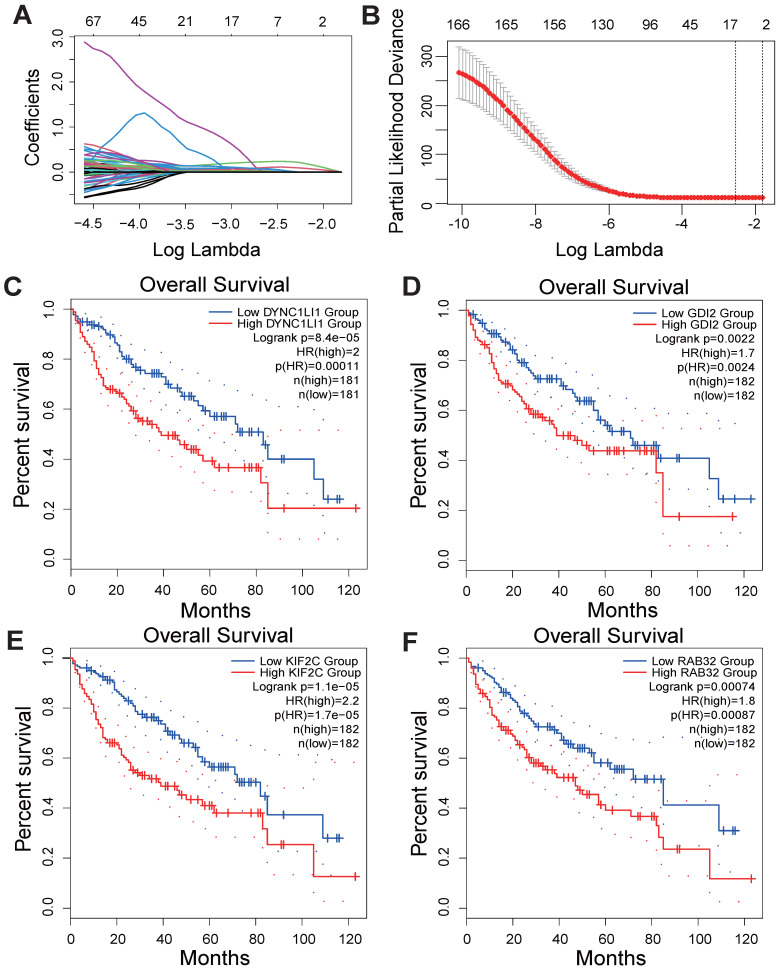
Development of a VMTRGs-based prognostic model. (A) LASSO coefficient spectrum of 166 candidate prognosis-related genes. Each curve shows the change in a gene's coefficient as the log lambda increases. (B) Cross-validation of adjustment parameter selection in a proportional hazards model. (C-F) Monogenic survival curves of four prognosis-related VMTRGs, DYNC1LI1 (C), GDI2 (D), KIF2C (E), and RAB32 (F), in the constructed risk model.

As shown in the flowchart of this study (Figure 1**)**, a total of 166 candidate prognosis-related genes for LIHC were first screened from 722 VMTRGs by using univariate Cox regression analysis (Table S3). To determine the top candidate VMTRGs, LASSO Cox regression analysis was performed to establish the VMTRGs-based signature. Subsequently, an optimal prognostic model containing four VMTRGs with nonzero coefficients was constructed (Figure 2A, B). The risk score was calculated by the following formula:

RC=0.2369744×DYNC1LI1+0.0438852×GDI2+0.118721×KIF2C+0.0355885×RAB32

Gene Expression Profiling Interactive Analysis 2 (GEPIA2) was used to determine the survival curves of the top 4 VMTRGs. The results showed that DYNC1LI1, GDI2, KIF2C, and RAB32 were hazard factors in this model, and elevated expression of these four VMTRGs was strongly associated with shorter survival in LIHC patients (Figure 2C-F).

**Figure 3:**
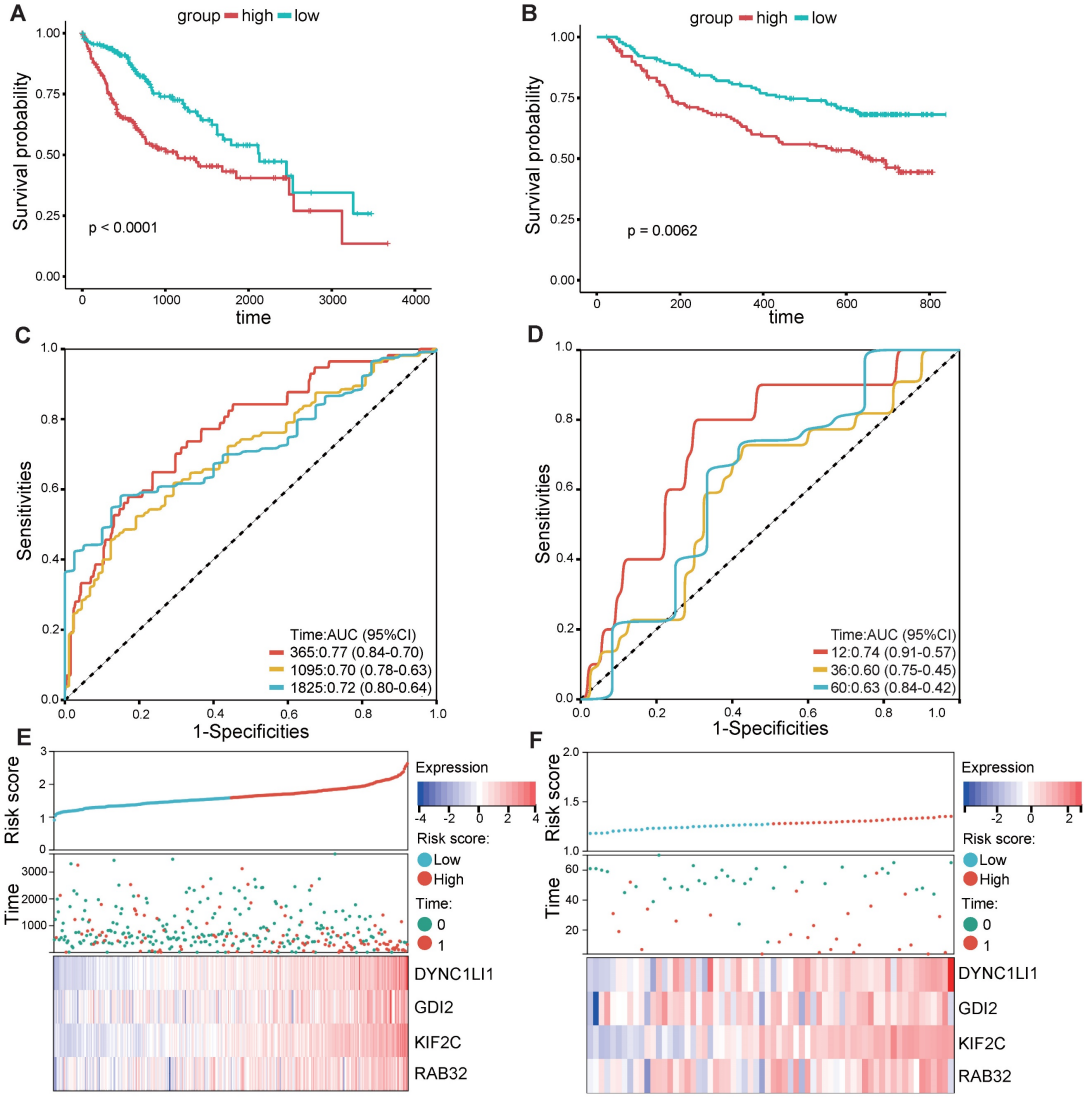
Validation of VMTRGs-based prognostic model. (A, B) Survival analysis of the high- and low-risk groups determined by VMTRGs-based prognostic model in the TCGA dataset (A) and GEO GPL13158 dataset (B). (C, D) Time-dependent ROC curves determining the prediction potential of the constructed risk model in the TCGA dataset (C) and GEO GPL13158 dataset (D). (E, F) The risk score, survival status and expression profile of four prognosis-related VMTRGs in the TCGA (E) and GEO GPL13158 (F) cohorts.

According to the median risk score, LIHC patients in the TCGA database were categorized into high- and low-risk groups. K‒M survival analysis of patients in the TCGA dataset revealed that the survival probability of the high-risk group was worse than that of the low-risk group (Figure 3A). To assess the robustness of this risk model derived from the TCGA dataset, GPL13158 microarray data from the GEO database were used for external validation. Again, poor prognostic outcomes were observed in the high-risk group in the validation cohort (Figure 3B), consistent with the results from the TCGA training cohort (Figure 3A). Then, receiver operating characteristic (ROC) curves were plotted to assess the predictive accuracy of the prognostic model. The areas under the curve (AUCs) for 1, 3, and 5 years in the TCGA cohort were 0.77, 0.70, and 0.72, respectively (Figure 3C). The area under the curve (AUC) for 1-, 3-, and 5-year PFS in the GEO-GPL13158 cohort was 0.74, 0.60, and 0.63, respectively (Figure 3D). The distributions of risk score, survival time, survival status and expression pattern of the four VMTRGs in the TCGA and GEO cohorts were also determined (Figure 3E, F). The results showed that elevated expression of four VMTRGs was significantly associated with short survival time and high death rate. These data collectively suggest that the LIHC-VMTRG signature has great prognostic potential.

### The mRNA and protein expression profiles of VMTRGs in LIHC

**Figure 4:**
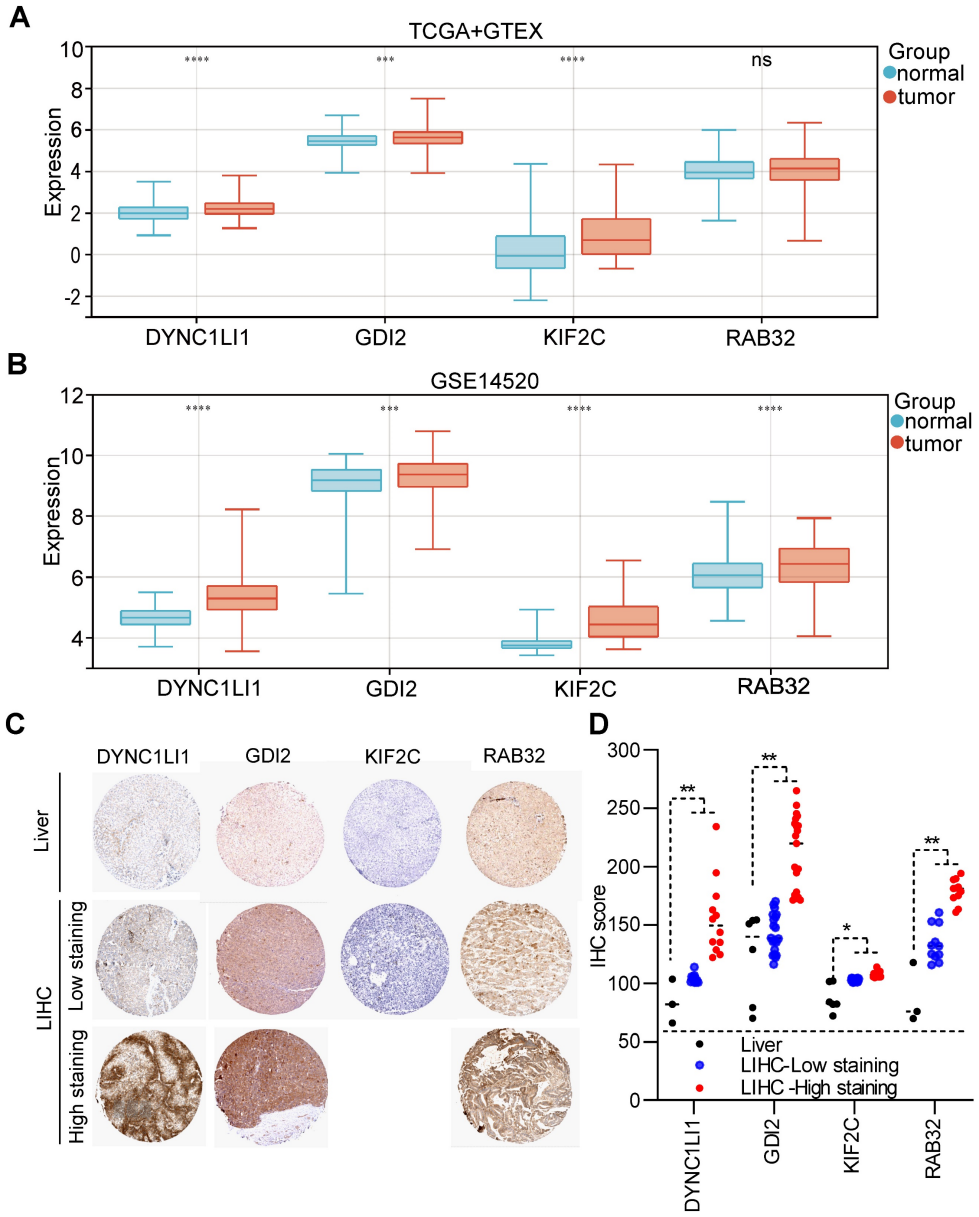
Expression levels of four prognosis-related VMTRGs in LIHC. Differential expression of DYNC1LI1, GDI2, KIF2C, and RAB32 between LIHC tissues and normal tissues. The sequencing data were extracted from the TCGA and GTEx databases (A) and the GEO dataset GSE14520 (B). (C) Representative IHC staining of DYNC1LI1, GDI2, KIF2C, and RAB32 in tissue microarrays obtained from the Human Protein Atlas. (D) IHC quantification of protein expression in liver and LIHC samples. *P<0.05, **P<0.01, ***P<0.001, ****P<0.0001, ns: no significant difference.

According to the TCGA+GTEx dataset, the mRNAs of three VMTRGs (DYNC1LI1, GDI2 and KIF2C) were highly expressed in LIHC tissues, while the RAB32 level did not differ between LIHC and normal tissues (Figure 4A). In contrast, in the validation dataset (GSE14520), the mRNA levels of all four VMTRGs (DYNC1LI1, GDI2, KIF2C and RAB32) were significantly upregulated in LIHC tissues (Figure 4B). Immunohistochemical (IHC) staining was used to investigate the protein expression of hub VMTRGs in liver and LIHC tissues. IHC staining data of these four VMTRGs were derived from the Human Protein Atlas (HPA) and a reference 23, and the results showed that the expression level of DYNC1LI1, GDI2, KIF2C and RAB32 were significantly higher in LIHC tissues (Figure 4C, 4D. Figure S1). All these data suggest that prognosis-related VMTRGs may be powerful diagnostic biomarkers for LIHC patients.

### Univariate and multivariate Cox analyses of the VMTRGs signature

**Figure 5:**
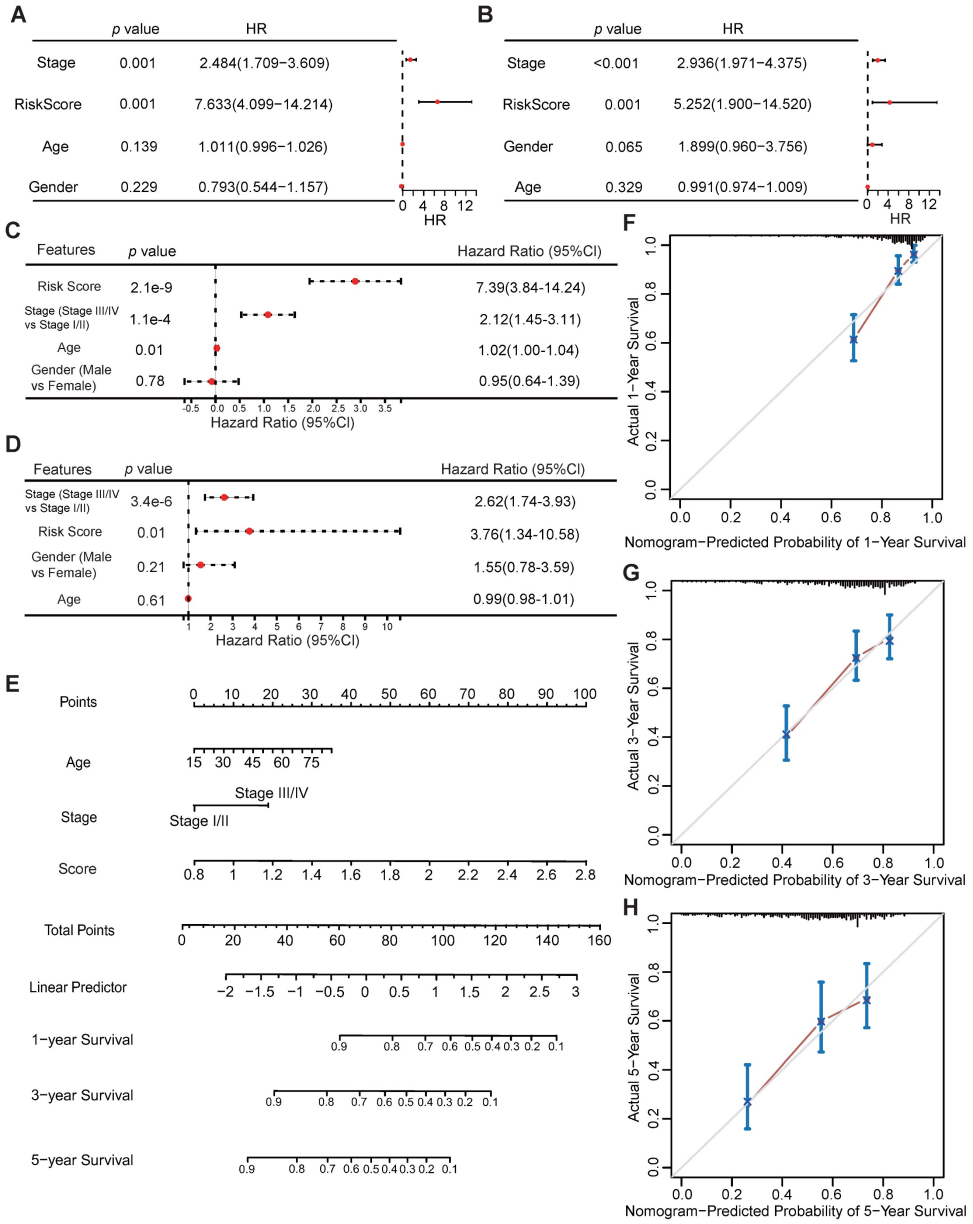
Potential of VMTRGs-based risk score, clinical factor, and survival prediction nomogram for predicting survival in patients with LIHC. (A, C) Univariate (A) and multivariate (C) Cox regression analyses were used to analyze the correlation between OS and the main clinical variables, including risk score, stage, age and sex, from the TCGA database. (B, D) Univariate (B) and multivariate (D) Cox regression analyses were performed to analyze the associations between OS and various clinical variables, including risk score, stage, age and sex, from the GEO database. (E) Alignment diagram model for predicting the OS of LIHC patients at 1, 3, and 5 years. (F-H) Calibration plots of the nomogram for 1- (F), 3- (G), and 5- (H) year survival.

Univariate and multivariate Cox regression analyses were performed to assess whether the VMTRGs-based signature is an independent prognostic predictor for LIHC patients. The patients' clinical information and risk scores were collected (Table S1). Univariate Cox regression analysis of the TCGA datasets showed that tumor stage and the VMTRGs-based risk score were found to be independent prognostic factors for LIHC patients (HR=2.484, *p*<0.001; HR=7.633, *p*<0.001) (Figure 5A). Cox multivariate regression analysis further showed that tumor stage and the VMTRGs-based risk score were found to be independent prognostic factors for LIHC patients (HR=2.12, *p*<0.001; HR=7.39, *p*<0.001) (Figure 5C). Furthermore, Gene Expression Omnibus (GEO) datasets were used to assess this VMTRGs-based signature. Both univariate and multivariate Cox analyses revealed that tumor stage (HR=2.936, *p*<0.001; HR=2.62, *p*<0.001) and VMTRGs risk score (HR=5.252, *p*<0.001; HR=3.76, *p*=0.01) were two independent prognostic factors for LIHC patients (Figure 5B, D).

### Construction and evaluation of the nomogram

Next, a nomogram containing VMTRGs, stage, and age was constructed to further assess the predictive power of the VMTRGs for individual OS outcomes. It can predict the probability of an exact outcome for any patient at a given time point. The ability of the nomogram to predict 1-, 3-, and 5-year OS outcomes in LIHC patients were calculated (Figure 5E). Subsequently, calibration plots were drawn to determine the reliability of the nomogram. The calibration plots showed that the effectiveness of this nomogram was highly accurate (Figure 5F-H). Thus, the selected VMTRGs are potential prognostic biomarkers for LIHC patients.

### Immune microenvironment analysis

The abundance of infiltrating immune cells in tumor tissues significantly affects the response to immunotherapy, suggesting that the immune microenvironment is a critical target for clinical treatment 24. Therefore, we assessed the association of the VMTRGs-based risk score with the immune microenvironment in LIHC tissues.

**Figure 6:**
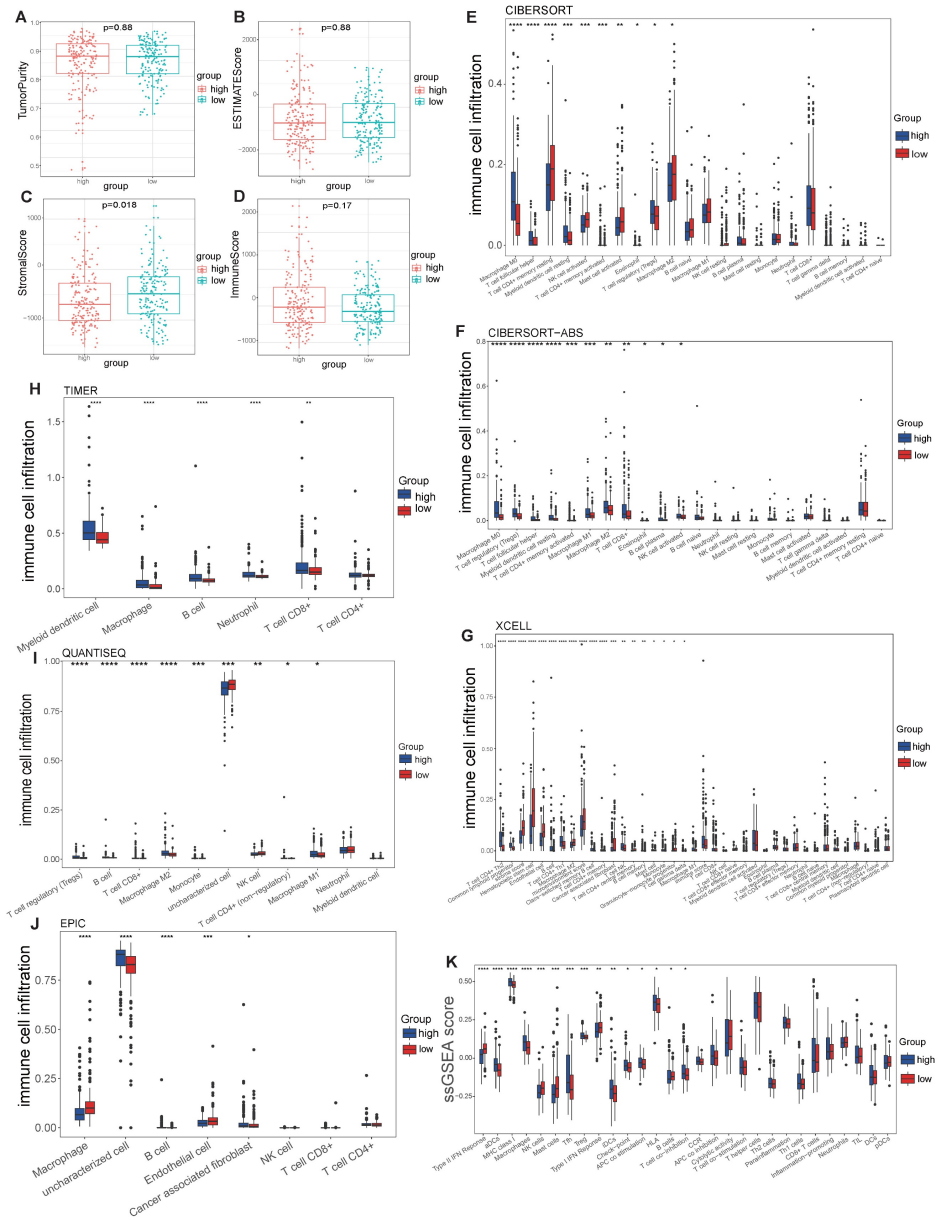
Correlation of the VMTRGs-based signature with immune cell infiltration in the TCGA cohort. (A, B) Tumor purity fractions and estimated scores for the high- and low-risk groups. (C, D) Differences in stromal and immune scores between the two risk groups. (E-J) Differences in immune cell infiltration abundance between the two risk groups. CIBERSORT (E), CIBERSORT-ABS (F), xCELL (G), TIMER (H), QUANTISEQ (I), and EPIC (J). (K) ssGSEA scores of immune function between the high- and low-risk groups. *P<0.05, **P<0.01, ***P<0.001, ****P<0.0001.

**Figure 7:**
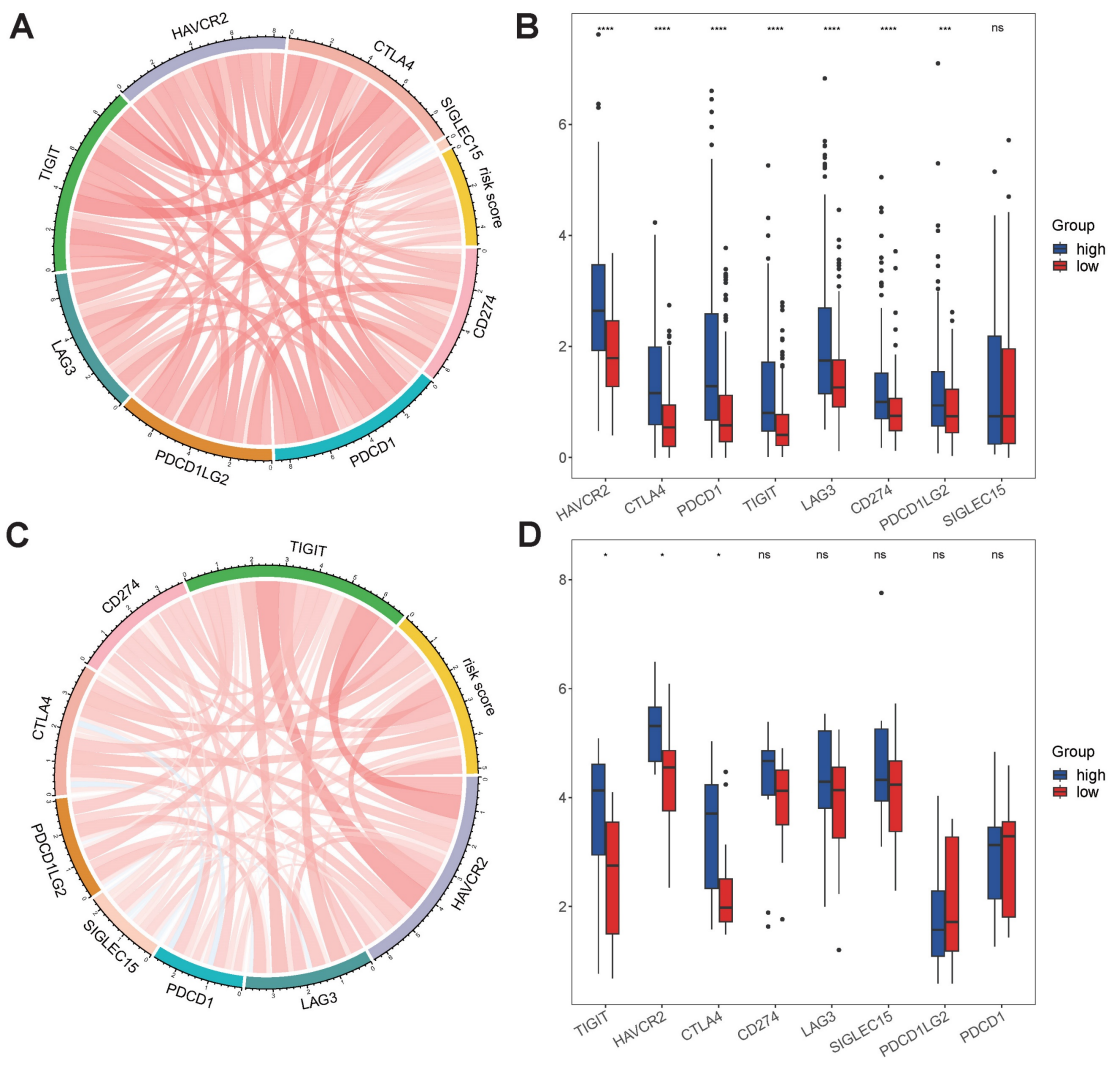
Immunotherapy response predicted by the VMTRGs-based risk score model. (A, C) Correlation analysis of the VMTRGs-based risk score with the expression of immune checkpoint molecules in the TCGA-LIHC (A) and GEO-GPL84402 (C) cohorts. (B) The mRNA levels of HAVCR2, CTLA4, CD274, PDCD1, PDCD1LG2, TIGIT and LAG3 were greater in the high-risk group in the TCGA-LIHC cohort. (D) The mRNA levels of TIGIT, HAVCR2, CTLA4 and SIGLEC15 were greater in the high-risk group in the GEO-GPL84402 dataset.

No differences in tumor purity, ESTIMATE score or immune score were observed between the high- and low-risk groups (Figure 6A, B, D). However, the stromal scores were significantly greater in the low-risk group (Figure 6C). Immunofunctional analysis revealed that aDCs, MHC complex class I macrophages, Tfh cells, Tregs and iDCs were highly active in the high-risk group, while type II IFN responses, NK cells, mast cells and type I IFN responses were more active in the low-risk group (Figure 6K). To further assess the association of the VMTRGs-based risk score with the immune microenvironment, we constructed different immune cell profiles in LIHC tissues by using six algorithms (CIBERSORT, CIBERSORT-ABS, xCELL, QUANTISE, TIMER and EPIC) (Figure 6E-J). High infiltration of M0-, M1- and M2- macrophages; CD8^+^ and follicular helper T cells; resting myeloid dendritic cells; Tregs; class-switched memory B cells; and neutrophils in LIHC tissues was frequently observed in the high-risk group. In contrast, enrichment of resting memory CD4^+^ T cells, activated NK cells and endothelial cells in LIHC tissues was more significant in the low-risk group. Furthermore, we found that high-risk scores were closely correlated with high expression of immune checkpoint molecules in the TCGA and GEO cohorts (Figure 7A, C). Notably, HAVCR2, CTLA4, CD274, PDCD1, PDCD1LG2, TIGIT and LAG3 were highly expressed in the high-risk group from the TCGA cohort (Figure 7B), whereas the expression of these checkpoint molecules was only slightly upregulated in the high-risk group from the GEO cohort (Figure 7D). Patients in high-risk groups may benefit from immunotherapies if treated with antibodies targeting these seven checkpoints. These results suggest that quantification via the VMTRGs-based risk score is highly valuable for LIHC patients and that the selected VMTRGs may be a powerful biomarker for clinical immunotherapy.

### Biological functional enrichment analysis

**Figure 8:**
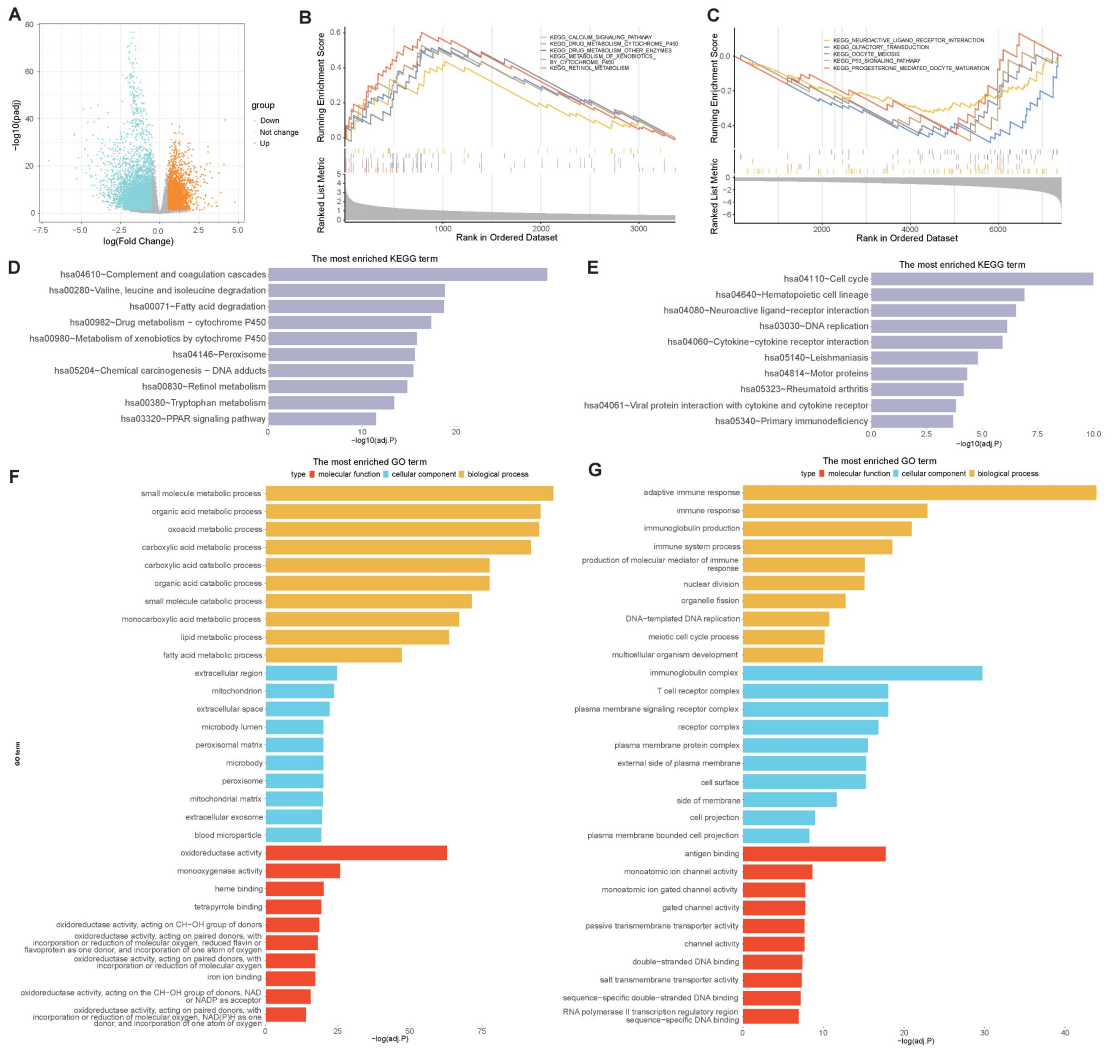
Biological process enrichment and functional analysis based on the VMTRGs-based risk score model. (A) Volcano map of differentially expressed VMTRGs in the TCGA-LIHC cohort. (B, C) GSEA of the high- (B) and low- (C) risk groups. (D, E) KEGG analysis of the high- (D) and low- (E) risk groups. (F, G) GO enrichment analyses of the high- (F) and low- (G) risk groups.

To determine the molecular characteristics of the high- and low-risk groups, distinct DEGs were first screened. Differential analyses were performed by using the DESeq2 package in R, and the differential expression was calculated when |logFC|> 0.5 and adj.P.Val.< 0.05. A total of 10802 DEGs were identified between the high- and low-risk groups, with 3370 upregulated and 7432 downregulated DEGs. A volcano map was subsequently drawn to visualize the DEG distribution between the two groups (Figure 8A). GSEA pathway analyses revealed that, in the high-risk group, the DEGs were predominantly enriched in the calcium signaling pathway, drug metabolism cytochrome P450 pathway, and other enzymes involved in retinol metabolism (Figure 8B), while the DEGs in the low-risk group were predominantly enriched in neuroactive ligand receptor interaction, olfactory transduction, oocyte meiosis, the p53 signaling pathway and progesterone-mediated oocyte maturation (Figure 8C). KEGG analyses revealed that DEGs in the high-risk group were highly enriched in complement and coagulation cascades, leucine and isoleucine degradation peroxisome, and PPAR signaling pathways (Figure 8D), while genes related to the cell cycle, DNA replication and cytokine-cytokine receptor interactions were enriched in the low-risk group (Figure 8E). In addition, GO functional analyses revealed that DEGs in the high-risk group were enriched in a series of metabolic processes, including small molecule, organic acid, and catabolic processes such as carboxylic acid and extracellular exosomes (Figure 8F), while DEGs in the low-risk group were enriched in several immune pathways, such as immune response and immune system processes (Figure 8G). These results indicate that the selected VMTRGs essentially contribute to many important biological functions of LIHC.

### Response to chemotherapeutics

**Figure 9:**
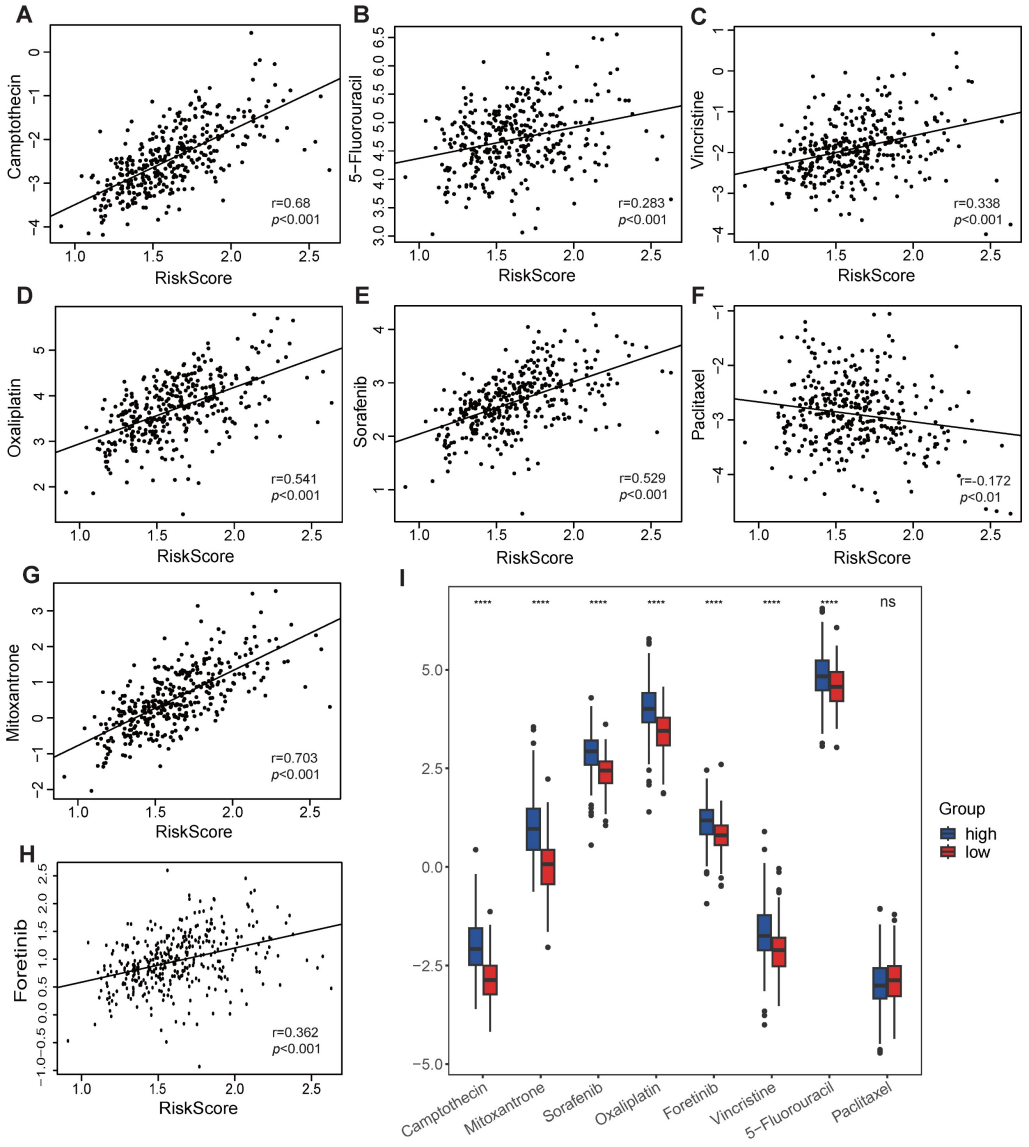
Association of the VMTRGs-based risk score with drug sensitivity. (A-H) The VMTRG-based risk score was correlated with the sensitivity (IC50) to camptothecin (A), 5-fluorouracil (B), vincristine (C), oxaliplatin (D), sorafenib (E), paclitaxel (F), mitoxantrone (G), and foretinib (H) in the TCGA-LIHC cohort. (I) Differences in drug sensitivity between the high- and low-risk groups. ****P<0.0001. ns: no significant difference.

As VMTRGs-based risk scores are correlated with poor outcomes in LIHC patients, anticancer drug sensitivity was further investigated in the VMTRGs signature-categorized cohort. Notably, significantly greater IC50s of camptothecin, 5-fluorouracil, vincristine, oxaliplatin, sorafenib, mitoxantrone, and foretinib were observed in the high-risk group (Figure 9). This phenomenon suggested that high-risk LIHC patients were highly resistant to these chemotherapies. In contrast, low- and high-risk LIHC patients were both sensitive to paclitaxel. These results may be highly important for drug selection during clinical treatment.

### Biological significance of GDI2 in LIHC progression

**Figure 10:**
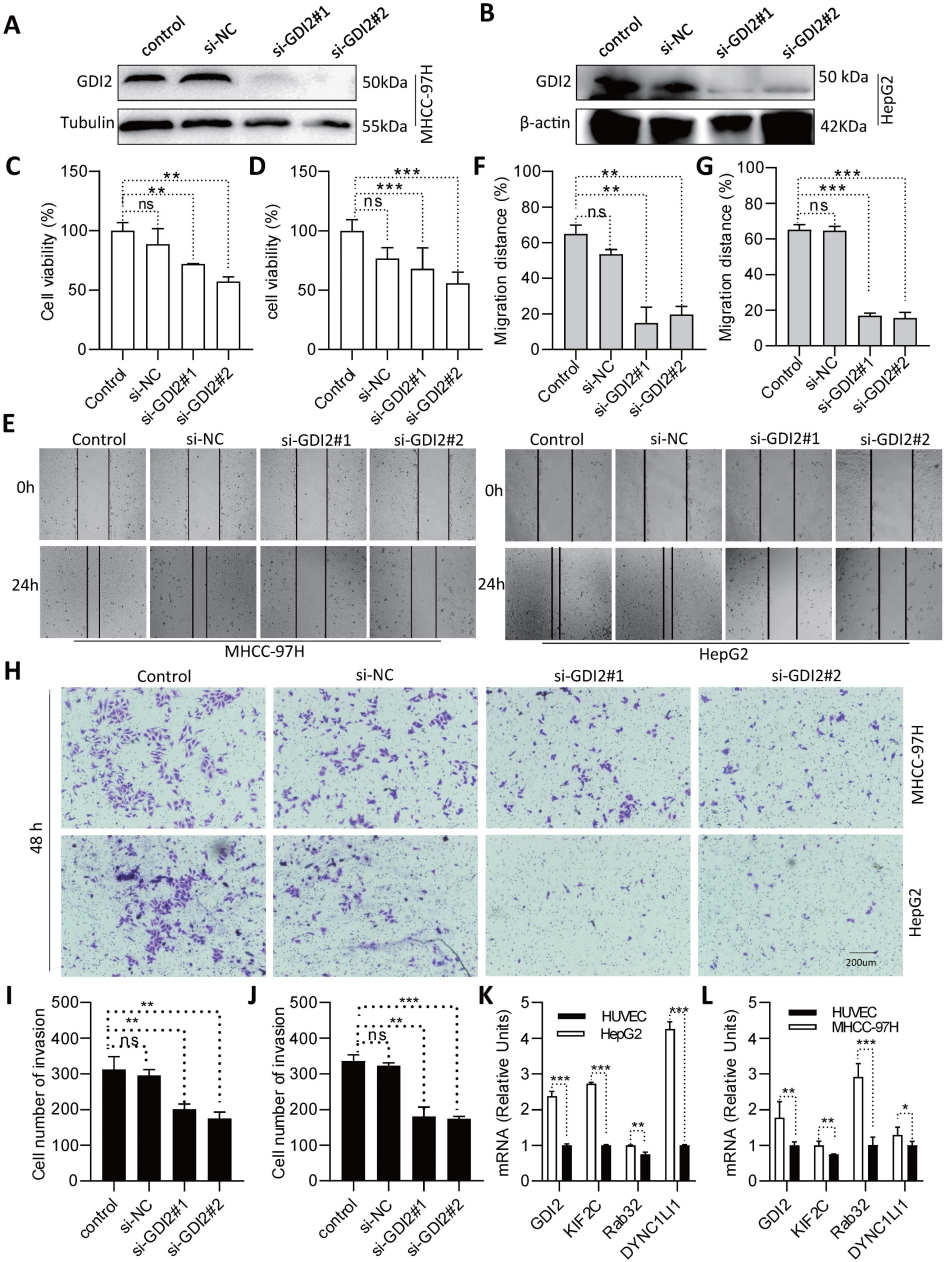
Identification of GDI2 biological function and expression and in LIHC cells. (A, B) GDI2 knockdown in MHCC-97H and HepG2 cells was determined by Western blotting. (C, D) Effect of GDI2 knockdown on MHCC-97H and HepG2 cell proliferation was determined by CCK-8 assays. (F, G) The effect of GDI2 knockdown on MHCC-97H and HepG2 cellular migration was determined by wound healing assays. Representative images are shown in (E). (I, J) The effect of GDI2 knockdown on MHCC-97H and HepG2 cellular invasion was determined by transwell assays. A representative image is shown in (H). (K, L) Determining the mRNA level of GDI2, DYNC1LI1, KIF2C, and RAB32 by Q-PCR. *P<0.05, **P<0.01, ***P<0.001, NS: no significant difference.

To further assess the role of the selected VMTRGs in LIHC, *in vitro* biological function experiments were conducted. Considering that GDI2 is a key regulator of Ras-mediated signaling, which critically affects the process of interorganellar vesicle transport, GDI2 was chosen for further investigation of its biological significance. After knocking down GDI2 expression (Figure 10A, B), the proliferation potential of MHCC-97H and HepG2 cells was strongly inhibited (Figure 10C, D). Furthermore, wound healing assays showed that GDI2 knockdown significantly inhibited the migration of MHCC-97H and HepG2 cells (Figure 10E-G). Notably, the cellular invasion capacity was also significantly decreased when GDI2 was knocked down (Figure 10H-J). Quantitative real-time PCR results showed that the genes of GDI2, DYNC1LI1, KIF2C, and RAB32 were highly expressed in MHCC-97H and HepG2 cells (Figure 10K, L. Figure S2-3). Taken together, these data suggest that GDI2 plays a critical role in controlling the proliferation, migration, and invasion of LIHC cells, which needs to be comprehensively investigated in the future.

## Discussion

The prognosis and treatment of LIHC are limited by the need for an accurate risk model. While liver transplantation, local ablation, chemoembolization and targeted therapies are available for LIHC, the clinical outcome remains modest 25. Researchers have used various signatures, including DNA repair-related genes, RNA modification-related genes, cell death-related genes, ligand‒receptor pair-related genes, copy number alteration-related lncRNAs, microenvironment-related lncRNAs and exosome-related lncRNAs, to predict the prognosis of LIHC 26-31. However, more precise prognostic signatures are still lacking. In this study, we conducted bioinformatics analyses to reveal a VMTRGs-based signature for LIHC. We established a VMTRGs-based risk model and demonstrated its feasibility for prognostic prediction and decision-making in LIHC patients. We further identified four hub genes (GDI2, DYNC1LI1, KIF2C, and RAB32) as prognostic biomarkers for LIHC. We also revealed the strong association of VMTRGs-based signature with immune microenvironment in LIHC. Additionally, we demonstrated the role of GDI2 in promoting the malignant phenotypes of LIHC cells. These investigations highlight the prognostic potential of VMTRGs-based signature and risk model.

Early studies have defined how hepatocytes maintain apical-basolateral membrane trafficking by polar vesicle-mediated transport, and how this process might be compromised in liver diseases 32. In mammalian cells, >60 RAB GTPases are known to be involved in vesicle trafficking, most of which are dysregulated in liver tissue 33. There are still active efforts underway toward determining the functions of these RAB GTPases 34. Since clarifying the collaborative functions of VMTRGs requires further exploration, investigating their prognostic potential could directly lead to clinical benefit. Until recently, the gene regulatory network controlling the whole vesicle transport process has not been well characterized. Apart from RAB family members, other VMTRGs are also critical for vesicle targeting/docking/fusion in cancer cells. As aberrant vesicle trafficking drives the development and progression of cancers, identifying reliable biomarkers from the perspective of VMTRGs could open a new avenue for early prognosis and effective treatment of LIHC patients.

Dysregulated expression of VMTRGs has been reported in several cancers, and the prognostic significance of VMTRGs in colorectal cancer (CRC), hepatocellular carcinoma (HCC) and lung adenocarcinoma (LUAD) has been preliminarily established. However, we constructed a new risk score model for LIHC based on the four key VMTRGs (DYNC1LI1, GDI2, KIF2C, and RAB32) (Figure 5). This finding is distinct from that of Lin Xin's report, in which only two VMTRGs (KIF2C and RAC1) were incorporated into the prognostic signature of HCC 23. Moreover, other laboratories disclosed 5 key VMTRGs (CNIH1, KIF20A, GALNT2, GRIA1, and AP3S1), 13 key VMTRGs (FOXD1, NXPH4, ADAD2, COX8C, C8G, CREG2, MUCL3, PSCA, NTF4, LRP2, INSL4, UGT2B4, and PLA2G2F) and 4 VMTRGs (GDI1, LMANL2, KLC3, and LRP2) as prognostic signatures for LUAD and CRC 6, 35, 36. Such variation may be due to the input number of identified VMTRGs used in different works: 85, 97 and 71 prognosis-associated VMTRGs were included for constructing risk models for LUAD, HCC and CRC, respectively. Compared to these reported VMTRGs-based risk models, our new risk model has good predictive ability, as the Cox regression analyses and the nomogram evaluation both proved its efficacy and accuracy in predicting the prognosis of LIHC patients (Figure 5).

In fact, four main differences exist between our work and previous works: (1) The source and composition of the datasets are different. We used the Reactome database to extract VMTRGs, whereas others used MSigDB for extracting MTRGs 36. (2) The methodologies for model construction and analysis were different. A protein-protein interaction (PPI) network, weighted gene co‑expression network analysis (WGCNA), copy number variation (CNV) and tumor mutational burden (TMB) were used to determine the gene expression patterns in clinical samples 35, 36. Such integration can only slightly benefit the original design. For categorizing clinical samples, the algorithms used in previous works, such as nonnegative matrix factorization (NMF) clustering, unsupervised clustering analysis, and consensus clustering analysis 23, 36, may not fit to our model construction and correlation analysis. (3) The immune analysis methods and indicators used differ. We did not use the immunophenoscore (IPS) or tumor immune dysfunction and exclusion (TIDE) score for immunotherapy evaluation, as described in other reports 35. This may explain the inconsistency of the immune infiltration data between our study and others. (4) Regarding gene enrichment analysis, the cell cycle, neuroactive ligand-receptor interactions and P53 signaling were not found to be related to patient survival in our work. This may be because the grouping methods used are distinct from others 6, 23, 35, 36.

Notably, based on our new VMTRGs signature, five biological pathways were dominantly enriched in high-risk LIHC patients (Figure 8). Calcium is an essential signal messenger that drives oncogenesis, favors metabolic reprogramming and gene expression in tumorigenesis. Many genes of calcium signaling (Figure 8B) are frequently mutated in LIHC. The deregulated Ca^2+^ homeostasis contributes to tumorigenesis, formation of metastasizing cells, and evasion of LIHC cell death 37, 38. Drug metabolism cytochrome P450 pathway (Figure 8B) plays key role in metabolizing xenobiotics and cancer drugs. Upregulation of cytochrome P450 frequently promotes the carcinogenesis process, treatment outcomes, and cancer drug resistance of LIHC 39, 40. Complement and coagulation cascades (Figure 8D) act as the connection between innate and adaptive immunity. Recent studies report that complement system can influence LIHC progression by regulating the tumor microenvironment, tumor cells, and cancer stem cells 41. As an important signaling molecules, peroxisome proliferator-activated receptors (PPARs) are involved in many physiological processes (Figure 8D), and can improve non-alcoholic fatty liver disease by regulating lipid metabolism. Recent studies report that PPARs can participate in the occurrence and development of LIHC by regulating metabolic pathways 42. Extracellular exosomes (Figure 8F) regulate the occurrence and development of LIHC via multiple processes. Studies report that exosomes promote LIHC progress by providing energy, transmitting protumor signals, regulating proangiogenic and propermeability factors, and inducing epithelial-mesenchymal transformation.

In immune microenvironment analysis, several important immune components are closely associated with LIHC progression. The highly infiltration of macrophages, Tfh, Regulatory T cells (Tregs), CD8^+^ T cells, resting myeloid dendritic cells, class-switched memory B cells and neutrophils were frequently observed in the high-risk group (Figure 6K, E-J). Infiltrating stromal, immune cells and extracellular matrix make up pernicious microenvironment of solid tumors. Increasing studies have investigated the interaction and crosstalk between stromal components and neoplastic cells. Evidences shows that stromal cells support the high mobility and metastasis potential of tumor cells by remodeling ECM or activating the intracellular signaling pathway 43. Tregs are universally recognized as a suppressor of immune system. With the ability of disturbing cytokine release and metabolism, they allow peripheral tolerance and immune escaping of tumor. Tregs harbor higher level of CD25 than that in effector T cells, thus acquire a high affinity with IL-2. This prevents normal combination of IL-2 and effector T cells, inhibiting immune response mediated by IL-2 44. Tregs can also be attracted by macrophages and tumor cell-expressed chemokines 45, which may explain why Tregs are enriched in poor-prognostic LIHC. High expressions of immune checkpoint molecules were also observed to closely associate with VMTRGs-based high-risk scores (Figure 7). Immune checkpoint is another pathway for tumor to escape immune attack. Tumor cells take advantage of checkpoint molecules' “STOP” signal to attenuate T cell activation 46. This well explains the close correlation between immune checkpoint activation and high-risk tumors.

According to our new VMTRGs-based risk model, GDI2, DYNC1LI1, KIF2C, and RAB32 were four hazard factors, and elevated expression of these VMTRGs was associated with shorter survival in LIHC patients (Figure 2C-F). As an accessory component of the dynein 1 complex, DYNC1LI1 plays a role in vesicle trafficking, chromosome segregation, and centrosome integrity. High expression of DYNC1LI1 promotes the progression, migration, and chemoresistance of colon cancer 47, 48. Kinesin family member 2 C (KIF2C) is frequently involved in MEK/ERK, mTOR, Wnt/β-catenin, P53 and TGF-β1/Smad signaling, immune infiltration, and DNA damage repair in tumorigenesis. Upregulation of KIF2C was shown to promote tumor cell migration, invasion, and chemotherapy resistance and inhibit DNA damage repair 49. RAB32 is expressed in many secretory epithelial cells and functions as a regulator of cellular metabolism by supporting mTORC1 signaling 50. A recent study revealed that it promotes glioblastoma migration and invasion via regulation of ERK/Drp1-mediated mitochondrial fission 23. In the current work, we focused on Rab-GDI2 function because it contributes to vesicle shuttling by regulating the activity of RAB GTPases. Rab-GDI2 regulates the GDP/GTP exchange of RAB proteins by inhibiting the dissociation of GDP and the subsequent binding of GTP 51. A latest report shows that Rab-GDI2 is a target of paclitaxel that affects the tumorigenesis of prostate cancer 52. Another report claims that Rab-GDI2 can be applied as a predictive biomarker for the diagnosis and prognosis of liver cancer 53. In the present study, we identified GDI2 as a powerful biomarker for the diagnosis and prognosis of LIHC (Figure 4, Figure 5) and revealed its ability to promote the proliferation, migration and invasion of LIHC cells (Figure 10).

Overall, a highlight of this study is the use of new VMTRG-based risk model for predicting the prognostic outcomes of LIHC patients. Based on the risk score determined by VMTRGs-based signature, high-risk LIHC patients are resistant to chemotherapeutics but benefit from partial ICIs. Risk stratification of LIHC patients by our new model can efficiently improve patient prognosis and treatment decisions. Future works should investigate the LIHC-promotion role of the identified four key VMTRGs by knockdown and rescue experiments both at cellular level and in mice model.

## Supplementary Material

Supplementary figures and tables.

## Figures and Tables

**Table 1:** The primers used in siRNA-mediated knockdown and real-time PCR.

Reactions	Gene name	Sequence
**RNA interference**		
	GDI2 #1	5'-UAUAAAGCAGCAUCUUAACCAGCUG-3'
	GDI2 #2	5'-CAAACAAUCCCAUUAGGCUAGAUGC-3'
**Q-PCR**		
	GDI2	5′-AAAAACGTCGCTTCAGGAAATTC-3′
		5′-AAAGTGCAAGAGCATGACCAG-3′
	DYNC1LI1	5′-CAGCAGGGTGGGATAATGATAAG-3′
		5′-AGTTGGTGGTTGCTTTGCTAA-3′
	KIF2C	5′-CTCAGTTCGGAGGAAATCATGTC-3′
		5′-TGCTCTTCGATAGGATCAGTCA-3′
	Rab32	5′-CAGGTGGACCAATTCTGCAAA-3′
		5′-GGCAGCTTCCTCTATGTTTATGT-3′
	GAPDH	5′- GGTGAAGGTCGGTGTGAACG-3′
		5′- CTCGCTCCTGGAAGATGGTG-3′
