# Supplementary material for: Vesicle-mediated transport-related genes predict the prognosis and immune microenvironment in hepatocellular carcinoma
Source: J Cancer. 2024 May 13;15(12):3645–62. doi: 10.7150/jca.94902 (PMC11190757; doi:10.7150/jca.94902)
Supplement: Supplementary file 1 — Supplementary figures and tables. [file jcav15p3645s1.pdf]

Table S1. Clinical information of LIHC and normal tissue samples

LIHC Samples	OS_status	os_time	Age	Gender	Stage	Risk
TCGA-G3-A25X	0	1779	73	male	Stage II	1.915839504
TCGA-DD-AADS	0	474	63	male	Stage I	1.163920163
TCGA-G3-AAV3	0	412	58	female	Stage II	1.537534941
TCGA-2Y-A9GU	0	1939	55	female	Stage I	1.267306832
TCGA-2Y-A9HB	0	260	66	male	Stage I	1.515527331
TCGA-DD-A73E	0	44	66	male	Stage I	1.426302653
TCGA-UB-A7MB	0	601	24	male	Stage II	2.010288703
TCGA-DD-A73A	0	728	71	male	Stage I	1.34892171
TCGA-DD-AAEG	0	719	59	female	Stage I	1.588205638
TCGA-ED-A806	1	56	50	female	Stage IIIA	1.863927664
TCGA-G3-AAV7	0	361	38	male	Stage II	2.573781702
TCGA-CC-A7IJ	0	382	56	male	Stage II	2.470384126
TCGA-2Y-A9H9	0	697	70	male	Stage I	1.581938235
TCGA-CC-5261	1	97	44	male	Stage II	2.082725424
TCGA-RC-A7SH	0	468	42	male	Stage II	1.500373469
TCGA-DD-A1EI	0	183	46	male	Stage I	1.912792163
TCGA-G3-A3CJ	0	594	52	male	Stage II	1.168307601
TCGA-GJ-A9DB	1	67	68	male	Stage I	1.744373958
TCGA-ED-A7PY	0	390	20	female	Stage II	1.430902406
TCGA-K7-A5RG	0	519	66	male	Stage I	1.665296912
TCGA-G3-A7M5	0	447	76	male	Stage I	1.249903132
TCGA-DD-AADN	0	898	59	male	Stage I	1.667910587
TCGA-CC-A8HS	1	300	18	male	Stage IIIC	1.94857138
TCGA-WQ-AB4B	0	395	62	male	Stage II	1.344671814
TCGA-FV-A23B	1	1852	70	female	Stage II	1.632778193
TCGA-FV-A3R3	1	366	38	female	Stage I	1.296203958
TCGA-G3-AAV0	0	476	58	male	Stage I	1.127562655
TCGA-5C-A9VG	0	328	58	male	Stage II	1.704806961
TCGA-DD-AAVV	0	2455	56	male	Stage II	1.719817613
TCGA-DD-AADB	0	1242	51	male	Stage I	1.952501394
TCGA-DD-AAVQ	0	2728	38	male	Stage I	1.647116563
TCGA-CC-5258	1	129	48	male	Stage II	2.076521505
TCGA-EP-A3JL	0	303	76	male	Stage I	1.433203866
TCGA-DD-A73C	0	701	65	female	Stage IIIA	1.568768061
TCGA-G3-AAV1	1	359	51	male	Stage IIIC	1.689674987
TCGA-DD-AAEE	0	810	55	male	Stage I	1.671186244
TCGA-G3-A25T	0	1553	45	female	Stage IIIA	1.866630376
TCGA-LG-A9QC	0	425	48	male	Stage I	1.405331272
TCGA-ED-A82E	0	408	60	female	Stage IIIA	1.560515702
TCGA-DD-AACF	1	365	68	male	Stage I	1.756957
TCGA-QA-A7B7	0	94	48	male	Stage II	2.043310116
TCGA-DD-AAC9	0	347	51	male	Stage I	1.290672979

TCGA-DD-AAE6	0	141	59	female	Stage I	1.602577573
TCGA-DD-A3A2	1	2131	76	female	Stage I	1.128277877
TCGA-DD-A39Y	1	171	67	male	Stage I	2.199810824
TCGA-CC-A3MB	1	315	36	male	Stage IIIA	1.983218829
TCGA-DD-AAE1	0	552	52	male	Stage I	1.3821855
TCGA-EP-A3RK	0	363	73	male	Stage IIIA	1.832116977
TCGA-G3-A3CG	0	673	80	male	Stage I	1.529973706
TCGA-DD-AA3A	1	410	81	female	Stage I	2.070701612
TCGA-DD-AACK	0	9	70	male	Stage I	1.721977571
TCGA-XR-A8TE	0	925	16	male	Stage IIIA	1.519552872
TCGA-G3-A25V	0	860	68	male	Stage I	1.45834418
TCGA-DD-A4NA	0	1008	67	female	Stage IIIC	1.796807044
TCGA-DD-AAD3	0	1295	43	male	Stage I	1.55148135
TCGA-DD-A118	0	3437	77	female	Stage II	1.337677508
TCGA-ED-A459	0	910	47	male	Stage II	1.918323736
TCGA-RC-A7SB	0	588	53	male	Stage II	1.432064267
TCGA-DD-AAEA	0	575	65	male	Stage I	1.516444323
TCGA-G3-A7M7	0	361	65	male	Stage I	1.45114624
TCGA-DD-AADP	0	458	45	male	Stage I	1.574695998
TCGA-DD-AACT	0	1562	69	female	Stage I	1.261195134
TCGA-UB-A7MA	0	848	62	female	Stage II	2.094286472
TCGA-DD-A73D	0	693	68	female	Stage II	1.347603113
TCGA-DD-A4NB	0	989	25	male	Stage I	1.454484004
TCGA-2Y-A9GT	1	1624	51	male	Stage I	1.371352749
TCGA-DD-AADL	0	636	58	male	Stage I	1.641958314
TCGA-DD-A1EG	1	1372	76	male	Stage I	1.701939018
TCGA-DD-A4ND	0	2746	56	female	Stage I	1.795476329
TCGA-2Y-A9H6	0	357	68	female	Stage I	1.333875243
TCGA-DD-AAW1	0	1989	55	male	Stage IIIA	1.107282806
TCGA-DD-A1EA	0	2415	68	male	Stage II	1.65899739
TCGA-ED-A66Y	1	296	51	female	Stage IIIA	1.817242005
TCGA-DD-AACH	1	195	69	male	Stage II	2.225780854
TCGA-XR-A8TC	0	1339	43	female	Stage I	1.926042528
TCGA-DD-A3A4	1	612	37	male	Stage IIIA	1.321784865
TCGA-DD-AADF	1	115	64	female	Stage I	1.492403844
TCGA-UB-AA0V	0	314	69	female	Stage I	1.283899783
TCGA-MR-A520	0	229	58	male	Stage I	1.18850077
TCGA-RC-A6M5	0	15	20	female	Stage IVA	1.18007779
TCGA-EP-A2KA	1	627	52	female	Stage IIIA	1.854636128
TCGA-DD-AAE7	0	644	72	male	Stage I	1.302037309
TCGA-DD-AAE4	0	608	49	female	Stage I	1.53253648
TCGA-DD-A4NF	0	942	72	male	Stage I	1.361955647
TCGA-DD-AACP	0	415	64	male	Stage I	1.942620461
TCGA-DD-AAE0	0	555	45	female	Stage IIIA	1.863163561

TCGA-BC-A8YO	0	562	66	female	Stage IIIC	2.021617987
TCGA-DD-A3A9	1	931	64	female	Stage IVB	1.495147569
TCGA-CC-A7II	0	399	54	male	Stage IIIA	2.295582725
TCGA-DD-AAD1	0	564	51	female	Stage I	1.763267718
TCGA-XR-A8TF	1	693	74	male	Stage I	1.624837443
TCGA-G3-A6UC	0	671	65	male	Stage IIIB	1.281498521
TCGA-DD-A1ED	0	2301	68	male	Stage I	1.193900964
TCGA-CC-5262	1	103	67	male	Stage IIIC	1.902444678
TCGA-ZS-A9CF	0	2412	64	male	Stage II	1.599316795
TCGA-FV-A3I1	1	247	81	female	Stage II	1.829576998
TCGA-DD-AAW0	0	2015	54	male	Stage I	1.542200145
TCGA-DD-AAD8	0	1219	73	female	Stage I	1.448360175
TCGA-RC-A7SK	0	472	59	male	Stage I	1.497009898
TCGA-DD-AAC8	1	16	72	male	Stage I	1.584097039
TCGA-GJ-A3OU	0	879	59	male	Stage I	1.796241645
TCGA-KR-A7K8	0	906	57	male	Stage I	1.656953432
TCGA-CC-A1HT	1	101	50	male	Stage IIIA	2.379927016
TCGA-BC-A69H	0	444	64	male	Stage II	1.968369881
TCGA-DD-AAVR	0	2513	44	male	Stage I	1.342866394
TCGA-DD-AAE3	0	566	50	male	Stage I	1.296159771
TCGA-DD-A4NN	1	899	56	female	Stage I	1.819409392
TCGA-DD-AACC	1	1685	61	male	Stage I	1.691028193
TCGA-DD-AADI	0	1085	43	female	Stage I	1.683733953
TCGA-DD-AAEB	0	478	60	male	Stage I	0.911669886
TCGA-2Y-A9H1	1	1229	58	male	Stage I	1.199004635
TCGA-DD-AACB	0	2324	74	female	Stage I	1.766274149
TCGA-WX-AA46	0	756	61	male	Stage II	1.215664462
TCGA-DD-A1EJ	1	1005	71	female	Stage IIIC	2.046046418
TCGA-EP-A2KB	1	596	46	female	Stage I	1.599877398
TCGA-ED-A66X	0	406	35	male	Stage IIIA	1.772917612
TCGA-DD-AACW	0	1424	43	male	Stage I	1.609163281
TCGA-NI-A4U2	1	1791	71	male	Stage IIIA	1.302230752
TCGA-ED-A97K	0	6	54	male	Stage IIIA	1.774436999
TCGA-G3-A3CK	0	585	61	male	Stage I	1.52217701
TCGA-DD-AADW	0	587	48	male	Stage I	2.013615519
TCGA-DD-AAVP	0	2752	48	male	Stage I	1.547177762
TCGA-DD-A39W	1	827	29	female	Stage III	1.455090449
TCGA-DD-A4NR	1	9	85	female	Stage I	1.85684467
TCGA-DD-AACU	0	1567	59	male	Stage I	1.738751399
TCGA-KR-A7K7	0	951	61	female	Stage II	1.661724478
TCGA-DD-AACQ	1	432	50	male	Stage II	1.341453341
TCGA-DD-A11B	1	14	73	male	Stage I	1.450917888
TCGA-DD-A4NS	1	2456	61	female	Stage I	1.531726976
TCGA-DD-A1EH	0	1495	23	male	Stage III	1.817030081

TCGA-DD-AAW3	0	1633	69	male	Stage I	1.347458202
TCGA-DD-AADA	0	1233	66	female	Stage I	1.043126288
TCGA-UB-A7MF	1	214	56	male	Stage IIIA	1.689171765
TCGA-DD-A119	1	223	40	male	Stage IV	1.488999691
TCGA-K7-A6G5	0	512	66	male	Stage I	1.471232341
TCGA-WJ-A86L	0	345	68	female	Stage I	1.370459944
TCGA-ZS-A9CG	0	341	55	male	Stage II	1.228770625
TCGA-O8-A75V	0	538	54	male	Stage I	1.486588944
TCGA-ED-A7PZ	0	6	61	male	Stage II	1.578636484
TCGA-DD-A1EL	1	415	23	male	Stage II	2.16206632
TCGA-G3-A5SL	0	621	70	male	Stage II	1.369124849
TCGA-DD-A1EE	1	349	73	male	Stage IIIA	1.744089695
TCGA-CC-5263	1	129	35	male	Stage IIIA	2.140070178
TCGA-G3-A3CI	0	180	71	male	Stage I	1.193507246
TCGA-ED-A5KG	0	854	60	female	Stage II	1.987891495
TCGA-DD-AAVS	0	1823	56	male	Stage I	1.685878622
TCGA-DD-A4NO	0	2245	65	male	Stage I	1.500617177
TCGA-CC-A3MA	1	303	61	male	Stage IIIA	2.089471826
TCGA-NI-A8LF	0	799	74	male	Stage I	1.336333822
TCGA-5C-A9VH	0	322	70	male	Stage I	1.59906285
TCGA-DD-A4NE	1	660	75	female	Stage IIIA	1.869914351
TCGA-FV-A4ZQ	0	12	52	male	Stage I	1.971756203
TCGA-K7-A5RF	0	631	64	male	Stage I	1.270025791
TCGA-DD-A1EB	0	2017	72	female	Stage I	1.41766242
TCGA-DD-AAVU	0	2202	46	male	Stage II	1.567894383
TCGA-DD-AADU	0	554	60	male	Stage II	1.148723763
TCGA-2Y-A9HO	0	3675	49	male	Stage IIIA	1.846943055
TCGA-GJ-A6CO	1	31	75	female	Stage II	1.770580475
TCGA-ED-A4XI	0	819	58	male	Stage II	1.332284813
TCGA-G3-A7M8	0	430	31	male	Stage I	1.179080976
TCGA-CC-A3M9	1	300	45	male	Stage IIIA	2.629437264
TCGA-FV-A2QR	1	581	75	male	Stage I	1.5582304
TCGA-MR-A8JO	0	330	34	male	Stage I	1.336827618
TCGA-DD-A3A6	1	3258	72	female	Stage II	1.208135983
TCGA-G3-AAV4	1	27	83	female	Stage I	1.675323418
TCGA-DD-A11D	1	1560	57	female	Stage I	1.37402053
TCGA-BD-A3EP	0	409	75	female	Stage I	1.656950481
TCGA-EP-A12J	0	570	62	male	Stage I	1.459134726
TCGA-4R-AA8I	1	262	66	male	Stage II	1.592355718
TCGA-CC-A7IF	1	649	59	male	Stage IIIA	1.301522746
TCGA-KR-A7KO	1	65	65	male	Stage I	1.432328069
TCGA-CC-A5UE	1	272	48	male	Stage IIIB	2.238187093
TCGA-HP-A5MZ	1	91	78	male	Stage I	1.449708982
TCGA-CC-A8HU	1	344	39	female	Stage IIIA	2.120444985

TCGA-DD-AAD0	0	453	55	male	Stage I	1.615569888
TCGA-DD-AAD5	0	1345	54	male	Stage I	2.003621299
TCGA-LG-A6GG	0	387	79	female	Stage II	1.529784458
TCGA-G3-A5SI	1	768	44	male	Stage II	1.843132811
TCGA-RG-A7D4	0	1098	69	male	Stage II	1.778866387
TCGA-KR-A7K2	0	829	64	male	Stage I	1.334964487
TCGA-DD-AADM	1	12	58	male	Stage II	1.63425563
TCGA-XR-A8TG	0	898	58	male	Stage I	1.783317784
TCGA-DD-AACD	1	381	48	male	Stage I	1.401287038
TCGA-3K-AAZ8	0	396	65	male	Stage IIIB	1.518650317
TCGA-DD-AADO	0	137	73	female	Stage I	1.466892042
TCGA-DD-AADJ	0	1066	70	female	Stage I	1.340201204
TCGA-ES-A2HS	1	688	80	male	Stage I	1.230354623
TCGA-ED-A7XP	0	400	53	female	Stage II	1.769445028
TCGA-G3-A7M9	1	56	70	male	Stage IIIB	2.539539028
TCGA-G3-A3CH	0	780	53	male	Stage IIIA	1.568998458
TCGA-G3-A25S	1	416	64	male	Stage I	1.793767458
TCGA-2Y-A9GW	1	1271	64	male	Stage I	1.439329019
TCGA-BC-A217	1	1397	75	female	Stage II	2.114905368
TCGA-DD-AACX	0	170	66	male	Stage II	1.731915019
TCGA-CC-A5UD	1	304	45	male	Stage IIIA	2.139812972
TCGA-EP-A26S	0	608	70	male	Stage I	1.425772177
TCGA-MI-A75E	0	507	61	male	Stage IIIC	1.463745416
TCGA-DD-AACO	0	1876	40	male	Stage I	1.528631323
TCGA-G3-A5SK	0	744	58	male	Stage I	1.199594493
TCGA-G3-A5SM	0	520	58	male	Stage II	1.485753914
TCGA-ED-A805	0	406	59	female	Stage IIIA	1.537533681
TCGA-FV-A3R2	1	194	75	male	Stage I	1.868127837
TCGA-DD-A116	1	1622	68	male	Stage IIIA	1.500913466
TCGA-DD-A39X	1	1694	78	female	Stage I	1.566265047
TCGA-2Y-A9H2	0	1731	64	female	Stage I	1.702648747
TCGA-CC-A9FS	0	211	55	male	Stage II	1.472702208
TCGA-XR-A8TD	0	1030	49	female	Stage IIIB	1.732692362
TCGA-DD-A4NK	1	1210	80	female	Stage IIIA	1.280707698
TCGA-FV-A4ZP	1	2486	78	male	Stage IIIA	1.768761845
TCGA-DD-AAE2	0	638	51	male	Stage I	1.542331636
TCGA-DD-A39Z	1	601	43	female	Stage II	1.57985849
TCGA-5R-AA1D	0	449	17	female	Stage IIIA	1.30659119
TCGA-2Y-A9H7	0	1168	81	female	Stage I	1.50778032
TCGA-CC-5264	1	102	71	male	Stage IIIA	2.279085302
TCGA-UB-A7ME	0	486	51	male	Stage I	1.710784869
TCGA-UB-A7MD	1	52	67	male	Stage I	1.688257997
TCGA-RC-A6M6	0	9	75	male	Stage II	2.184012673
TCGA-DD-A3A8	1	11	75	male	Stage II	1.168366703

TCGA-ED-A7PX	0	6	48	female	Stage II	1.915939085
TCGA-DD-AACZ	1	171	63	female	Stage I	2.224883175
TCGA-DD-A39V	1	643	77	male	Stage II	1.55366353
TCGA-ZS-A9CE	0	1241	79	female	Stage II	1.230760562
TCGA-5R-AA1C	0	520	57	male	Stage II	1.727736625
TCGA-G3-A25Z	0	655	58	male	Stage I	1.547979478
TCGA-5R-AAAM	1	46	65	female	Stage II	1.390671572
TCGA-ES-A2HT	1	438	54	male	Stage I	1.305624484
TCGA-DD-A3A7	1	419	67	male	Stage IIIB	1.709544981
TCGA-DD-AAE9	0	722	69	male	Stage I	1.363324029
TCGA-CC-A7IH	0	365	58	male	Stage IIIA	1.360252147
TCGA-DD-AACL	1	107	66	female	Stage I	2.212333063
TCGA-CC-5259	0	250	60	female	Stage IIIC	1.351395708
TCGA-G3-A7M6	0	632	60	female	Stage I	1.909259832
TCGA-DD-AAD6	0	672	66	male	Stage IIIA	1.563467534
TCGA-FV-A2QQ	0	729	80	male	Stage I	1.496942556
TCGA-2Y-A9GX	0	2442	68	male	Stage I	1.649494368
TCGA-BD-A3ER	0	1115	62	male	Stage II	1.337011339
TCGA-DD-AADC	1	425	53	male	Stage I	2.281857925
TCGA-BC-A10Z	1	34	62	female	Stage I	1.600398668
TCGA-5C-AAPD	0	20	61	male	Stage II	1.676409894
TCGA-DD-AAED	0	763	51	male	Stage I	1.483796236
TCGA-DD-AACI	0	1618	69	male	Stage II	1.599673483
TCGA-G3-A25Y	1	452	52	female	Stage I	1.929857867
TCGA-PD-A5DF	1	639	58	female	Stage IIIB	1.72490476
TCGA-DD-A114	1	1149	42	male	Stage II	2.334309331
TCGA-DD-AAVY	0	1970	56	male	Stage IIIA	1.299209307
TCGA-CC-A123	0	219	24	female	Stage IIIA	1.701319807
TCGA-DD-AADG	0	1145	70	male	Stage IIIA	1.27277318
TCGA-CC-A7IG	1	299	47	male	Stage II	2.142599622
TCGA-DD-A3A3	1	535	45	male	Stage I	1.417477167
TCGA-2Y-A9GZ	1	848	82	female	Stage II	1.28891817
TCGA-K7-AAU7	0	359	61	male	Stage II	1.942130028
TCGA-BW-A5NO	0	20	50	male	Stage IIIA	1.675898199
TCGA-DD-AAVX	0	1718	38	male	Stage II	1.33909467
TCGA-DD-A4NJ	0	928	54	female	Stage II	1.837182186
TCGA-DD-AACS	0	1804	39	male	Stage I	1.767594887
TCGA-UB-AA0U	0	327	60	male	Stage II	1.747099526
TCGA-CC-A9FW	0	248	68	male	Stage IIIA	1.656179236
TCGA-CC-A8HT	1	140	74	male	Stage IIIA	2.185190803
TCGA-G3-AAUZ	0	480	48	male	Stage I	1.485538415
TCGA-G3-A25U	0	1636	63	female	Stage I	1.631595364
TCGA-DD-AAEK	0	1067	51	male	Stage II	1.512037714
TCGA-DD-AAEH	0	784	73	male	Stage I	1.458407334

TCGA-DD-A73F	0	1085	77	female	Stage I	1.585872327
TCGA-2Y-A9HA	1	36	70	male	Stage II	1.663449432
TCGA-UB-A7MC	0	500	59	male	Stage IIIA	1.632328371
TCGA-DD-AACG	1	469	52	male	Stage II	1.818988704
TCGA-2Y-A9GY	1	757	64	female	Stage II	1.923956778
TCGA-DD-A1EF	1	394	57	female	Stage I	1.680878927
TCGA-DD-AADQ	0	436	59	male	Stage II	1.438413465
TCGA-DD-AACA	0	2301	65	male	Stage I	1.60719199
TCGA-DD-A1EK	1	558	64	female	Stage IVB	1.356076553
TCGA-DD-AAD2	0	658	66	male	Stage I	1.70292516
TCGA-DD-A11C	0	662	69	male	Stage I	1.411523821
TCGA-DD-AAEI	0	1531	72	male	Stage I	1.516148019
TCGA-G3-AAV5	0	354	67	male	Stage II	1.69659657
TCGA-DD-AADK	0	1049	68	female	Stage II	1.4590154
TCGA-2Y-A9H5	1	555	59	female	Stage I	1.523301596
TCGA-FV-A3I0	0	848	76	female	Stage II	1.704212892
TCGA-CC-A7IL	1	278	61	male	Stage IIIA	1.569113122
TCGA-DD-A115	1	2542	53	male	Stage IIIA	1.626653934
TCGA-BC-A69I	0	387	69	male	Stage I	1.226559624
TCGA-MI-A75C	0	291	64	male	Stage I	1.54457457
TCGA-DD-AADV	0	574	50	male	Stage I	1.832628722
TCGA-DD-AACV	0	1531	53	male	Stage I	1.847090131
TCGA-DD-AADR	0	2028	58	male	Stage I	1.621955636
TCGA-EP-A2KC	1	19	62	male	Stage I	1.441416139
TCGA-DD-AACJ	0	2102	75	male	Stage II	1.370758453
TCGA-LG-A9QD	0	366	68	male	Stage IIIA	1.172221645
TCGA-WX-AA44	0	615	64	female	Stage I	1.650617519
TCGA-DD-A113	0	2425	55	female	Stage II	1.659484423
TCGA-FV-A496	0	10	84	female	Stage I	1.323212352
TCGA-DD-A1EC	0	602	20	female	Stage I	1.753497458
TCGA-DD-AAVW	0	2317	35	male	Stage I	1.517051337
TCGA-2Y-A9H3	0	1516	45	male	Stage II	1.115799924
TCGA-DD-A4NH	0	917	65	female	Stage IIIB	1.816411779
TCGA-CC-A7IE	1	217	57	male	Stage IIIA	1.901534086
TCGA-DD-A4NQ	1	373	60	male	Stage II	1.873166767
TCGA-DD-A4NG	1	802	77	male	Stage IIIA	1.2706727
TCGA-DD-AAW2	0	1855	69	male	Stage I	1.425673697
TCGA-DD-A73B	1	283	72	female	Stage I	1.746398874
TCGA-DD-A4NI	0	816	67	male	Stage II	1.149492145
TCGA-MI-A75G	0	698	63	male	Stage II	1.618593843
TCGA-BC-A10X	1	770	52	female	Stage IIIA	1.388096111
TCGA-CC-A3MC	0	363	54	male	Stage IIIA	1.678780773
TCGA-2Y-A9GV	1	2532	54	female	Stage I	1.368243554
TCGA-DD-A73G	0	3478	73	female	Stage I	1.473869802

TCGA-CC-5260	1	87	61	female	Stage IIIC	2.144290033
TCGA-CC-A7IK	1	262	59	male	Stage IIIA	1.89100884
TCGA-DD-A4NL	0	1711	46	male	Stage I	1.183048588
TCGA-DD-A3A1	1	233	65	male	Stage IIIA	1.639282636
TCGA-G3-AAV2	0	372	50	male	Stage I	1.304860803
TCGA-BC-A216	0	1351	62	female	Stage IIIA	1.895912956
TCGA-DD-A4NV	0	2398	61	male	Stage IIIA	1.51161134
TCGA-ED-A627	0	423	74	male	Stage I	1.610930478
TCGA-RC-A7S9	0	640	47	female	Stage I	1.625446945
TCGA-DD-AADD	0	1231	51	male	Stage I	1.87120228
TCGA-BC-A3KG	0	680	68	female	Stage II	2.105143486
TCGA-DD-AACN	0	1302	32	male	Stage I	1.707226951
TCGA-G3-AAV6	1	65	53	female	Stage IIIA	1.858376821
TCGA-DD-AAVZ	0	1900	38	male	Stage I	1.685060968
TCGA-DD-AACE	0	2184	62	male	Stage I	1.20554215
TCGA-BC-A3KF	0	8	66	female	Stage I	1.52116853
TCGA-ZS-A9CD	1	1386	73	male	Stage II	1.412802853
TCGA-DD-AADY	0	555	55	female	Stage I	1.480524902
TCGA-2Y-A9H4	0	1452	68	male	Stage I	1.557698137
TCGA-WX-AA47	1	556	33	female	Stage IIIA	1.087306466
TCGA-G3-A5SJ	0	698	59	male	Stage I	1.825573123
TCGA-YA-A8S7	1	412	68	male	Stage IIIA	2.15546729
TCGA-FV-A495	0	1	51	female	Stage II	1.68916061
TCGA-DD-A4NP	0	3308	32	male	Stage I	1.185910463
TCGA-DD-A3A5	1	3125	66	female	Stage III	1.612747901
TCGA-DD-AACY	0	1450	61	male	Stage I	1.346475825
TCGA-CC-A5UC	1	347	63	male	Stage IIIA	1.935619584
TCGA-CC-A8HV	1	279	51	female	Stage II	1.844478123
TCGA-BC-A5W4	1	547	69	male	Stage IIIA	1.654861091
TCGA-RC-A7SF	0	579	66	male	Stage I	1.364170708
TCGA-RC-A6M4	0	22	74	female	Stage IIIA	1.560919954
TCGA-ED-A7XO	0	427	29	male	Stage IIIA	1.703912772

Normal Samples	Risk
GTEX-WZTO-0626-SM-4PQYY	0.840461
GTEX-12WSM-0726-SM-5GCOW	1.022758
GTEX-12WSI-0226-SM-5GCNA	1.075542
GTEX-12696-0826-SM-5EGGE	0.706518
GTEX-1212Z-0226-SM-59HLF	0.845232
GTEX-ZYT6-0626-SM-5E45V	0.552492
GTEX-T6MN-1226-SM-3NMA5	0.959738
GTEX-11DXZ-0126-SM-5EGGY	1.065773
GTEX-11UD2-1626-SM-5EQM3	1.128886
GTEX-147F4-1426-SM-5LUA8	0.958972
GTEX-144GM-1326-SM-5LU5E	0.949065

GTEX-13N11-0926-SM-5IJG2	1.619623
GTEX-12ZZZ-1326-SM-59HKW	0.427941
GTEX-RNOR-1426-SM-48FDJ	0.650479
GTEX-WFON-1726-SM-4LVMQ	0.9328
GTEX-VUSG-0126-SM-4KL1X	0.818594
GTEX-11ZUS-2526-SM-59872	0.524588
GTEX-QEG4-1826-SM-4R1JN	0.929328
GTEX-YB5E-0326-SM-5IFHU	1.745675
GTEX-O5YT-0826-SM-3TW8N	1.721976
GTEX-11TUW-1726-SM-5BC5C	0.941214
GTEX-11EQ9-0526-SM-5A5JZ	1.24234
GTEX-UPIC-0926-SM-4IHLV	1.391849
GTEX-ZVT4-0626-SM-5E45T	0.847331
GTEX-13NZ9-1326-SM-5MR3V	1.049967
GTEX-139UC-1426-SM-5IJGH	0.862267
GTEX-12KS4-1326-SM-5LUB3	1.318472
GTEX-14753-1626-SM-5NQ9L	0.490853
GTEX-11WQC-0726-SM-5EQMR	0.998275
GTEX-X4XY-1626-SM-46MVN	0.973602
GTEX-13PVR-0126-SM-5S2PY	0.953633
GTEX-RN64-1826-SM-48FDV	0.824891
GTEX-13FLV-0326-SM-5N9DJ	0.896959
GTEX-S33H-1626-SM-4AD68	1.062797
GTEX-11DXY-0526-SM-5EGGQ	0.846683
GTEX-13113-1326-SM-5GCOI	0.894073
GTEX-RU72-1426-SM-46MUF	0.898131
GTEX-13OVJ-1026-SM-5IFGI	1.175172
GTEX-13O3O-1826-SM-5IFGW	0.963241
GTEX-WYVS-1926-SM-4SOIC	1.219946
GTEX-ZPU1-0826-SM-57WG2	1.060542
GTEX-13SLX-1226-SM-5S2Q6	1.049694
GTEX-ZEX8-0826-SM-4WKHK	1.137729
GTEX-13FTW-1126-SM-5J2NV	1.110531
GTEX-13QJC-0726-SM-5RQJK	0.739559
GTEX-OOBJ-0826-SM-3NB2K	1.132481
GTEX-P78B-1326-SM-3P611	1.347178
GTEX-ZYY3-0626-SM-5NQ6W	0.982262
GTEX-14C38-1526-SM-5RQJ7	0.984881
GTEX-ZAB4-0826-SM-5LU9D	0.936584
GTEX-12WSL-0226-SM-5CVMJ	1.113476
GTEX-WY7C-0726-SM-4ONCB	1.424142
GTEX-YECK-1926-SM-4W21H	0.833572
GTEX-145LU-1326-SM-5LU9N	0.827659
GTEX-13NYB-1026-SM-5IFH3	1.016119

GTEX-RM2N-1926-SM-48FCU	1.11281
GTEX-X261-1726-SM-4PQYT	1.185611
GTEX-X4EP-1026-SM-4QAS5	0.898725
GTEX-11OF3-0726-SM-5BC4Z	0.878313
GTEX-11TT1-1726-SM-5EQLJ	1.47085
GTEX-147JS-1126-SM-5RQIW	0.921537
GTEX-139YR-0226-SM-5IFEM	1.108233
GTEX-Y5V5-0926-SM-4VBPZ	0.833217
GTEX-ZAB5-0426-SM-5CVMI	0.883724
GTEX-131XE-0326-SM-5LZVO	1.160995
GTEX-1497J-0726-SM-5Q5D1	1.26341
GTEX-RTLS-1326-SM-46MUN	0.892908
GTEX-1399R-1226-SM-5P9GF	1.153974
GTEX-ZF2S-3026-SM-4WWCH	1.183606
GTEX-13PVQ-1526-SM-5IFEQ	0.9171
GTEX-YFC4-1526-SM-5IFJS	0.967156
GTEX-1192X-1026-SM-5H12P	1.623443
GTEX-139TS-1426-SM-5IFJD	0.73946
GTEX-132AR-0426-SM-5IFH8	1.42263
GTEX-13OW6-2626-SM-5IFF2	0.839273
GTEX-ZTPG-1426-SM-51MT3	1.188473
GTEX-S32W-1926-SM-4AD63	1.377512
GTEX-QDVN-0826-SM-48TZ2	1.261708
GTEX-Y5LM-0426-SM-4VBRO	1.537743
GTEX-131YS-1626-SM-5HL6C	0.617785
GTEX-1399T-0826-SM-5IFES	0.941576
GTEX-131XH-0626-SM-5LZWH	0.740376
GTEX-XBEC-1526-SM-4AT68	0.853873
GTEX-11NUK-1226-SM-5P9GM	0.652983
GTEX-RWSA-1426-SM-47JXA	0.894411
GTEX-XXEK-1126-SM-4BRUX	1.106495
GTEX-UTHO-2426-SM-4JBHD	1.046672
GTEX-11ZTS-1426-SM-5EQMM	0.61439
GTEX-WQUQ-1926-SM-4OOSA	1.06111
GTEX-11NV4-1326-SM-5HL6V	0.754064
GTEX-U8XE-1526-SM-4E3HT	1.106758
GTEX-XOTO-0826-SM-4B65O	0.797776
GTEX-Z9EW-0426-SM-5CVM9	0.754679
GTEX-ZZPU-0426-SM-5GZYH	0.928828
GTEX-1269C-0626-SM-5FQSS	1.025588
GTEX-13VXU-0926-SM-5IFFH	0.838366
GTEX-P44G-1126-SM-3NM9D	0.747496
GTEX-WYVS-1926-SM-4PQZ2	1.173078
GTEX-14A5I-1726-SM-5QGQ5	0.829108

GTEX-11ZVC-0726-SM-5FQT9	0.785533
GTEX-13112-1426-SM-5EGH8	0.72285
GTEX-ZVT3-1626-SM-5GU66	0.933934
GTEX-SJXC-1226-SM-4DM78	0.892011
GTEX-139TU-0826-SM-5IJFG	0.866031
GTEX-Q2AG-1126-SM-48U1P	0.998649
GTEX-ZF29-2026-SM-4WWB7	1.021977
GTEX-13NZB-0626-SM-5IFH6	0.781488
GTEX-14E1K-0326-SM-5S2PE	0.860186
GTEX-ZVP2-0626-SM-51MSO	0.843679
GTEX-13FTZ-0726-SM-5IFFY	0.702517
N1	0.776605
N2	0.620576
N3	0.811088
N4	0.70065
N5	0.626164
N6	0.653308
N7	0.731443
N8	0.735363
N9	0.718011
N10	0.713122
N11	0.900791
N12	0.691175
N13	0.695628
N14	0.626145
N15	0.804068
N16	0.667418
N17	0.709689
N18	0.766327
N19	0.732862
N20	0.778611
N21	0.732042
N22	0.664528
N23	0.798005
N24	0.639353
N25	0.74987
N26	0.745232
N27	0.665785
N28	0.831417
N29	0.610188
N30	0.645054
N31	0.71207
N32	0.554047
N33	0.612905

N34	0.759954
N35	0.508079
N36	0.868854
N37	0.630718
N38	0.727003
N39	0.664263
N40	0.695676
N41	0.86667
N42	0.621412
N43	0.71284
N44	0.711699
N45	0.743698
N46	0.704741
N47	0.653767
N48	0.703716
N49	0.717069
N50	0.639798

Table S2. VMTRGs extracted from the Reactome genesets.

Origional Member	NCBI(Entrez) Gene Id	Gene Symbol
ENSG00000001626	1080	CFTR
ENSG00000003056	4074	M6PR
ENSG00000003393	57679	ALS2
ENSG00000004059	381	ARF5
ENSG00000004975	1856	DVL2
ENSG00000005243	51226	COPZ2
ENSG00000006125	163	AP2B1
ENSG00000006451	5898	RALA
ENSG00000007168	5048	PAFAH1B1
ENSG00000007255	79090	TRAPPC6A
ENSG00000008256	9265	CYTH3
ENSG00000009844	51534	VTA1
ENSG00000010327	23166	STAB1
ENSG00000010610	920	CD4
ENSG00000011405	5286	PIK3C2A
ENSG00000011454	23637	RABGAP1
ENSG00000019169	8685	MARCO
ENSG00000029534	286	ANK1
ENSG00000029725	9135	RABEP1
ENSG00000034713	11345	GABARAPL2
ENSG00000038945	4481	MSR1
ENSG00000041353	5874	RAB27B
ENSG00000042753	1175	AP2S1
ENSG00000047579	84062	DTNBP1

ENSG00000049245	9341	VAMP3
ENSG00000053501	55850	USE1
ENSG00000054116	27095	TRAPPC3
ENSG00000054523	23095	KIF1B
ENSG00000057608	2665	GDI2
ENSG00000063245	29924	EPN1
ENSG00000065609	9892	SNAP91
ENSG00000065882	23216	TBC1D1
ENSG00000066455	9950	GOLGA5
ENSG00000066735	26153	KIF26A
ENSG00000067715	6857	SYT1
ENSG00000068354	4943	TBC1D25
ENSG00000068796	3796	KIF2A
ENSG00000069974	5873	RAB27A
ENSG00000070182	6710	SPTB
ENSG00000070367	10640	EXOC5
ENSG00000070371	8218	CLTCL1
ENSG00000072134	22905	EPN2
ENSG00000072274	7037	TFRC
ENSG00000072958	8907	AP1M1
ENSG00000073060	949	SCARB1
ENSG00000073921	8301	PICALM
ENSG00000073969	4905	NSF
ENSG00000074319	7251	TSG101
ENSG00000074660	8578	SCARF1
ENSG00000074695	3998	LMAN1
ENSG00000075624	60	ACTB
ENSG00000075785	7879	RAB7A
ENSG00000075886	113457	TUBA3D
ENSG00000075945	22920	KIFAP3
ENSG00000076554	7163	TPD52
ENSG00000077380	1781	DYNC1I2
ENSG00000077549	832	CAPZB
ENSG00000078053	273	AMPH
ENSG00000078269	8871	SYNJ2
ENSG00000079462	5050	PAFAH1B3
ENSG00000079616	3835	KIF22
ENSG00000079805	1785	DNM2
ENSG00000080371	23011	RAB21
ENSG00000080824	3320	HSP90AA1
ENSG00000081014	23431	AP4E1
ENSG00000081479	4036	LRP2
ENSG00000083937	25978	CHMP2B
ENSG00000084674	338	APOB

ENSG00000084731	3797	KIF3C
ENSG00000084733	10890	RAB10
ENSG00000085063	966	CD59
ENSG00000085733	2017	CTTN
ENSG00000085832	2060	EPS15
ENSG00000086065	51510	CHMP5
ENSG00000086598	10959	TMED2
ENSG00000086827	9183	ZW10
ENSG00000087086	2512	FTL
ENSG00000088727	64147	KIF9
ENSG00000088986	8655	DYNLL1
ENSG00000089006	27131	SNX5
ENSG00000089177	55614	KIF16B
ENSG00000089818	25977	NECAP1
ENSG00000090889	24137	KIF4A
ENSG00000090989	55763	EXOC1
ENSG00000091513	7018	TF
ENSG00000092108	23256	SCFD1
ENSG00000092531	8773	SNAP23
ENSG00000093183	9117	SEC22C
ENSG00000095139	372	ARCN1
ENSG00000095383	55357	TBC1D2
ENSG00000099246	22931	RAB18
ENSG00000099940	9342	SNAP29
ENSG00000099992	83874	TBC1D10A
ENSG00000100055	27128	CYTH4
ENSG00000100077	157	GRK3
ENSG00000100083	26088	GGA1
ENSG00000100099	89781	HPS4
ENSG00000100196	11015	KDELR3
ENSG00000100228	9609	RAB36
ENSG00000100241	6305	SBF1
ENSG00000100266	11252	PACSIN2
ENSG00000100280	162	AP1B1
ENSG00000100342	8542	APOL1
ENSG00000100345	4627	MYH9
ENSG00000100478	11154	AP4S1
ENSG00000100528	10175	CNIH1
ENSG00000100599	79890	RIN3
ENSG00000100815	9321	TRIP11
ENSG00000100934	10484	SEC23A
ENSG00000101160	1522	CTSZ
ENSG00000101162	81027	TUBB1
ENSG00000101199	55738	ARFGAP1

ENSG00000101200	551	AVP
ENSG00000101246	10139	ARFRP1
ENSG00000101350	9371	KIF3B
ENSG00000101421	128866	CHMP4B
ENSG00000103051	25839	COG4
ENSG00000103111	22879	MON1B
ENSG00000103197	7249	TSC2
ENSG00000103365	23062	GGA2
ENSG00000103496	6810	STX4
ENSG00000103769	8766	RAB11A
ENSG00000104067	7082	TJP1
ENSG00000104164	26258	BLOC1S6
ENSG00000104671	10671	DCTN6
ENSG00000104833	10382	TUBB4A
ENSG00000104892	147700	KLC3
ENSG00000104915	8677	STX10
ENSG00000104946	79735	TBC1D17
ENSG00000105063	22870	PPP6R1
ENSG00000105186	84079	ANKRD27
ENSG00000105221	208	AKT2
ENSG00000105339	22898	DENND3
ENSG00000105355	10226	PLIN3
ENSG00000105402	8775	NAPA
ENSG00000105438	10945	KDELR1
ENSG00000105443	9266	CYTH2
ENSG00000105612	1777	DNASE2
ENSG00000105649	5864	RAB3A
ENSG00000105669	11316	COPE
ENSG00000105829	10282	BET1
ENSG00000106299	8976	WASL
ENSG00000106367	1174	AP1S1
ENSG00000106617	51422	PRKAG2
ENSG00000106636	10652	YKT6
ENSG00000106927	259	AMBP
ENSG00000106976	1759	DNM1
ENSG00000107021	54662	TBC1D13
ENSG00000107036	57589	RIC1
ENSG00000107185	9827	RGP1
ENSG00000107295	6456	SH3GL2
ENSG00000107521	3257	HPS1
ENSG00000107651	11196	SEC23IP
ENSG00000107862	8729	GBF1
ENSG00000108433	9570	GOSR2
ENSG00000108582	1362	CPD

ENSG00000108587	9527	GOSR1
ENSG00000108669	9267	CYTH1
ENSG00000108774	5878	RAB5C
ENSG00000108821	1277	COL1A1
ENSG00000108953	7531	YWHAE
ENSG00000109321	374	AREG
ENSG00000109686	152503	SH3D19
ENSG00000109861	1075	CTSC
ENSG00000109971	3312	HSPA8
ENSG00000110075	55291	PPP6R3
ENSG00000110169	3263	HPX
ENSG00000110195	2348	FOLR1
ENSG00000110395	867	CBL
ENSG00000110514	8567	MADD
ENSG00000111229	10094	ARPC3
ENSG00000111481	22818	COPZ1
ENSG00000111540	5869	RAB5B
ENSG00000111652	50813	COPS7A
ENSG00000111725	5564	PRKAB1
ENSG00000111731	9847	C2CD5
ENSG00000111737	11021	RAB35
ENSG00000111885	4121	MAN1A1
ENSG00000111907	7164	TPD52L1
ENSG00000112685	55770	EXOC2
ENSG00000112893	4124	MAN2A1
ENSG00000112984	10112	KIF20A
ENSG00000113070	1839	HBEGF
ENSG00000113140	6678	SPARC
ENSG00000113282	9685	CLINT1
ENSG00000113441	4012	LNPEP
ENSG00000113597	80006	TRAPPC13
ENSG00000113615	10802	SEC24A
ENSG00000113734	662	BNIP1
ENSG00000114251	7474	WNT5A
ENSG00000114270	1294	COL7A1
ENSG00000114354	10342	TFG
ENSG00000114745	64689	GORASP1
ENSG00000114988	81562	LMAN2L
ENSG00000115091	10096	ACTR3
ENSG00000115145	10254	STAM2
ENSG00000115306	6711	SPTBN1
ENSG00000115353	6869	TACR1
ENSG00000115561	51652	CHMP3
ENSG00000115592	53632	PRKAG3

ENSG00000115839	22930	RAB3GAP1
ENSG00000115977	22848	AAK1
ENSG00000116266	6814	STXBP3
ENSG00000116489	829	CAPZA1
ENSG00000116675	9829	DNAJC6
ENSG00000116711	5321	PLA2G4A
ENSG00000116852	23046	KIF21B
ENSG00000116903	149371	EXOC8
ENSG00000117020	10000	AKT3
ENSG00000117533	8674	VAMP4
ENSG00000117643	57134	MAN1C1
ENSG00000118004	78989	COLEC11
ENSG00000118137	335	APOA1
ENSG00000118473	84251	SGIP1
ENSG00000118508	10981	RAB32
ENSG00000118640	8673	VAMP8
ENSG00000118873	25782	RAB3GAP2
ENSG00000119396	51552	RAB14
ENSG00000119414	5537	PPP6C
ENSG00000119522	57706	DENND1A
ENSG00000119541	9525	VPS4B
ENSG00000119729	23433	RHOQ
ENSG00000120341	89866	SEC16B
ENSG00000120694	10808	HSPH1
ENSG00000120805	400	ARL1
ENSG00000121022	10987	COPS5
ENSG00000121542	26984	SEC22A
ENSG00000121621	81930	KIF18A
ENSG00000121634	2703	GJA8
ENSG00000121742	10804	GJB6
ENSG00000121743	2700	GJA3
ENSG00000121749	64786	TBC1D15
ENSG00000122126	4952	OCRL
ENSG00000122218	1314	COPA
ENSG00000122674	51622	CCZ1
ENSG00000122705	1211	CLTA
ENSG00000123240	10133	OPTN
ENSG00000123384	4035	LRP1
ENSG00000123416	10376	TUBA1B
ENSG00000123570	51209	RAB9B
ENSG00000123595	9367	RAB9A
ENSG00000123892	23682	RAB38
ENSG00000124222	8675	STX16
ENSG00000124333	6845	VAMP7

ENSG00000124507	29993	PACSIN1
ENSG00000124882	2069	EREG
ENSG00000125337	3834	KIF25
ENSG00000125447	23163	GGA3
ENSG00000125733	9322	TRIP10
ENSG00000125814	63908	NAPB
ENSG00000125875	128637	TBC1D20
ENSG00000126062	11070	TMEM115
ENSG00000126214	3831	KLC1
ENSG00000126895	554	AVPR2
ENSG00000127241	5648	MASP1
ENSG00000127328	117177	RAB3IP
ENSG00000127527	58513	EPS15L1
ENSG00000127824	7277	TUBA4A
ENSG00000127946	3092	HIP1
ENSG00000128245	7533	YWHAH
ENSG00000129083	1315	COPB1
ENSG00000129250	10749	KIF1C
ENSG00000129354	10053	AP1M2
ENSG00000129559	4738	NEDD8
ENSG00000130164	3949	LDLR
ENSG00000130203	348	APOE
ENSG00000130294	547	KIF1A
ENSG00000130340	51429	SNX9
ENSG00000130475	23149	FCHO1
ENSG00000130724	27243	CHMP2A
ENSG00000130787	9026	HIP1R
ENSG00000131233	81025	GJA9
ENSG00000131437	11127	KIF3A
ENSG00000131475	84313	VPS25
ENSG00000131558	60412	EXOC4
ENSG00000131791	5565	PRKAB2
ENSG00000131966	55860	ACTR10
ENSG00000132254	23647	ARFIP2
ENSG00000132405	57533	TBC1D14
ENSG00000132465	3512	JCHAIN
ENSG00000132612	27183	VPS4A
ENSG00000132669	54453	RIN2
ENSG00000132718	23208	SYT11
ENSG00000132842	8546	AP3B1
ENSG00000132912	51164	DCTN4
ENSG00000133103	57511	COG6
ENSG00000133138	54885	TBC1D8B
ENSG00000133812	81846	SBF2

ENSG00000134243	6272	SORT1
ENSG00000134262	10717	AP4B1
ENSG00000134265	8774	NAPG
ENSG00000134287	377	ARF3
ENSG00000134308	10971	YWHAQ
ENSG00000134594	9363	RAB33A
ENSG00000134644	9698	PUM1
ENSG00000134871	1284	COL4A2
ENSG00000134970	51014	TMED7
ENSG00000135018	29979	UBQLN1
ENSG00000135040	60560	NAA35
ENSG00000135218	948	CD36
ENSG00000135249	60561	RINT1
ENSG00000135355	84694	GJA10
ENSG00000135441	2647	BLOC1S1
ENSG00000135597	85021	REPS1
ENSG00000135677	2799	GNS
ENSG00000135720	1783	DYNC1LI2
ENSG00000135775	22796	COG2
ENSG00000135823	10228	STX6
ENSG00000135968	9648	GCC2
ENSG00000136011	55576	STAB2
ENSG00000136100	51028	VPS36
ENSG00000136111	9882	TBC1D4
ENSG00000136152	83548	COG3
ENSG00000136238	5879	RAC1
ENSG00000136240	11014	KDELR2
ENSG00000136631	11311	VPS45
ENSG00000136717	274	BIN1
ENSG00000136738	8027	STAM
ENSG00000136816	27348	TOR1B
ENSG00000136827	1861	TOR1A
ENSG00000136856	29988	SLC2A8
ENSG00000136874	55014	STX17
ENSG00000136883	113220	KIF12
ENSG00000136933	10244	RABEPK
ENSG00000136935	2800	GOLGA1
ENSG00000137100	11258	DCTN3
ENSG00000137145	55667	DENND4C
ENSG00000137171	89953	KLC4
ENSG00000137261	9856	KIAA0319
ENSG00000137267	7280	TUBB2A
ENSG00000137285	347733	TUBB2B
ENSG00000137486	408	ARRB1

ENSG00000137502	27314	RAB30
ENSG00000137807	9493	KIF23
ENSG00000137877	51332	SPTBN5
ENSG00000137942	54874	FNBP1L
ENSG00000138069	5861	RAB1A
ENSG00000138071	10097	ACTR2
ENSG00000138073	10113	PREB
ENSG00000138107	10121	ACTR1A
ENSG00000138160	3832	KIF11
ENSG00000138182	9585	KIF20B
ENSG00000138190	54536	EXOC6
ENSG00000138663	51138	COPS4
ENSG00000138674	22872	SEC31A
ENSG00000138760	950	SCARB2
ENSG00000138768	8615	USO1
ENSG00000138778	1062	CENPE
ENSG00000138798	1950	EGF
ENSG00000138802	10427	SEC24B
ENSG00000139116	55605	KIF21A
ENSG00000139722	79720	VPS37B
ENSG00000139977	122830	NAA30
ENSG00000140022	85439	STON2
ENSG00000140506	79748	LMAN1L
ENSG00000140600	6457	SH3GL3
ENSG00000140941	81631	MAP1LC3B
ENSG00000141030	8533	COPS3
ENSG00000141200	84643	KIF2B
ENSG00000141252	55275	VPS53
ENSG00000141367	1213	CLTC
ENSG00000141429	2589	GALNT1
ENSG00000141480	409	ARRB2
ENSG00000141551	1453	CSNK1D
ENSG00000141971	93343	MVB12A
ENSG00000141985	6455	SH3GL1
ENSG00000142192	351	APP
ENSG00000142208	207	AKT1
ENSG00000142765	84958	SYTL1
ENSG00000142945	11004	KIF2C
ENSG00000143545	5872	RAB13
ENSG00000143553	23557	SNAPIN
ENSG00000143641	2590	GALNT2
ENSG00000143761	375	ARF1
ENSG00000143786	149111	CNIH3
ENSG00000143858	127833	SYT2

ENSG00000143947	6233	RPS27A
ENSG00000143952	51542	VPS54
ENSG00000144524	64708	COPS7B
ENSG00000144566	5868	RAB5A
ENSG00000144635	51143	DYNC1LI1
ENSG00000144674	2803	GOLGA4
ENSG00000144747	7110	TMF1
ENSG00000144891	185	AGTR1
ENSG00000145362	287	ANK2
ENSG00000145979	51256	TBC1D7
ENSG00000146352	134829	CLVS2
ENSG00000146574	221960	CCZ1B
ENSG00000146648	1956	EGFR
ENSG00000146966	27147	DENND2A
ENSG00000147010	30011	SH3KBP1
ENSG00000147127	347517	RAB41
ENSG00000147457	91782	CHMP7
ENSG00000148248	6836	SURF4
ENSG00000148396	9919	SEC16A
ENSG00000148429	9712	USP6NL
ENSG00000149043	90019	SYT8
ENSG00000149182	84364	ARFGAP2
ENSG00000149428	10525	HYOU1
ENSG00000149823	738	VPS51
ENSG00000150527	4253	MIA2
ENSG00000150961	9871	SEC24D
ENSG00000150991	7316	UBC
ENSG00000151150	288	ANK3
ENSG00000151532	143187	VTI1A
ENSG00000151746	636	BICD1
ENSG00000151779	51594	NBAS
ENSG00000152056	130340	AP1S3
ENSG00000152086	112714	TUBA3E
ENSG00000152291	10618	TGOLN2
ENSG00000152661	2697	GJA1
ENSG00000152700	51128	SAR1B
ENSG00000153071	1601	DAB2
ENSG00000153130	60592	SCOC
ENSG00000153339	22878	TRAPPC8
ENSG00000154305	375056	MIA3
ENSG00000154710	27342	RABGEF1
ENSG00000154917	51560	RAB6B
ENSG00000155511	2890	GRIA1
ENSG00000155961	116442	RAB39B

ENSG00000155975	137492	VPS37A
ENSG00000155980	3798	KIF5A
ENSG00000157020	6396	SEC13
ENSG00000157107	115548	FCHO2
ENSG00000157191	55707	NECAP2
ENSG00000157978	26119	LDLRAP1
ENSG00000158270	81035	COLEC12
ENSG00000158560	1780	DYNC1I1
ENSG00000158623	26958	COPG2
ENSG00000159082	8867	SYNJ1
ENSG00000159210	11267	SNF8
ENSG00000159248	57369	GJD2
ENSG00000160216	56894	AGPAT3
ENSG00000160218	7109	TRAPPC10
ENSG00000160460	57731	SPTBN4
ENSG00000160654	917	CD3G
ENSG00000160948	51160	VPS28
ENSG00000161203	1173	AP2M1
ENSG00000161800	29127	RACGAP1
ENSG00000162065	57465	TBC1D24
ENSG00000162236	6811	STX5
ENSG00000162409	5563	PRKAA2
ENSG00000162704	10092	ARPC5
ENSG00000162777	79961	DENND2D
ENSG00000162849	55083	KIF26B
ENSG00000163235	7039	TGFA
ENSG00000163283	250	ALPP
ENSG00000163466	10109	ARPC2
ENSG00000163554	6708	SPTA1
ENSG00000163631	213	ALB
ENSG00000163808	56992	KIF15
ENSG00000164077	84315	MON1A
ENSG00000164265	117156	SCGB3A2
ENSG00000164292	22836	RHOBTB3
ENSG00000164411	375519	GJB7
ENSG00000164597	10466	COG5
ENSG00000164627	221458	KIF6
ENSG00000164692	1278	COL1A2
ENSG00000164695	92421	CHMP4C
ENSG00000164924	7534	YWHAZ
ENSG00000165006	51271	UBAP1
ENSG00000165115	55582	KIF27
ENSG00000165219	26130	GAPVD1
ENSG00000165474	2706	GJB2

ENSG00000165527	382	ARF6
ENSG00000165699	7248	TSC1
ENSG00000165912	29763	PACSIN3
ENSG00000166128	51762	RAB8B
ENSG00000166200	9318	COPS2
ENSG00000166444	6764	DENND2B
ENSG00000166557	23423	TMED3
ENSG00000166598	7184	HSP90B1
ENSG00000166685	9382	COG1
ENSG00000166747	164	AP1G1
ENSG00000166847	84516	DCTN5
ENSG00000166913	7529	YWHAB
ENSG00000167110	2801	GOLGA2
ENSG00000167286	915	CD3D
ENSG00000167291	125058	TBC1D16
ENSG00000167461	4218	RAB8A
ENSG00000167515	51693	TRAPPC2L
ENSG00000167552	7846	TUBA1A
ENSG00000167553	84790	TUBA1C
ENSG00000167632	83696	TRAPPC9
ENSG00000167702	90990	KIFC2
ENSG00000167987	55048	VPS37C
ENSG00000167994	5866	RAB3IL1
ENSG00000167996	2495	FTH1
ENSG00000168079	286133	SCARA5
ENSG00000168090	10980	COPS6
ENSG00000168092	5049	PAFAH1B2
ENSG00000168118	5867	RAB4A
ENSG00000168374	378	ARF4
ENSG00000168434	91949	COG7
ENSG00000168461	11031	RAB31
ENSG00000168538	60684	TRAPPC11
ENSG00000168542	1281	COL3A1
ENSG00000168685	3575	IL7R
ENSG00000168818	53407	STX18
ENSG00000169221	26000	TBC1D10B
ENSG00000169223	10960	LMAN2
ENSG00000169252	154	ADRB2
ENSG00000169562	2705	GJB1
ENSG00000169696	79058	ASPSCR1
ENSG00000169727	2873	GPS1
ENSG00000169891	9185	REPS2
ENSG00000170027	7532	YWHAG
ENSG00000170043	58485	TRAPPC1

ENSG00000170296	11337	GABARAP
ENSG00000170315	7314	UBB
ENSG00000170348	10972	TMED10
ENSG00000170456	160518	DENND5B
ENSG00000170471	57148	RALGAPB
ENSG00000170743	143425	SYT9
ENSG00000170759	3799	KIF5B
ENSG00000171853	51112	TRAPPC12
ENSG00000172007	83452	RAB33B
ENSG00000172780	339122	RAB43
ENSG00000173020	156	GRK2
ENSG00000173213	260334	TUBB8B
ENSG00000173230	2804	GOLGB1
ENSG00000173432	6288	SAA1
ENSG00000173744	3267	AGFG1
ENSG00000173898	6712	SPTBN2
ENSG00000173905	27333	GOLIM4
ENSG00000174485	10260	DENND4A
ENSG00000174791	9610	RIN1
ENSG00000174804	8322	FZD4
ENSG00000174808	685	BTC
ENSG00000174839	201627	DENND6A
ENSG00000174871	254263	CNIH2
ENSG00000174903	81876	RAB1B
ENSG00000174996	64837	KLC2
ENSG00000175203	10540	DCTN2
ENSG00000175416	1212	CLTB
ENSG00000175463	374403	TBC1D10C
ENSG00000175582	5870	RAB6A
ENSG00000175793	2810	SFN
ENSG00000175984	163259	DENND2C
ENSG00000176014	84617	TUBB6
ENSG00000176108	79643	CHMP6
ENSG00000176428	155382	VPS37D
ENSG00000176986	9632	SEC24C
ENSG00000177169	8408	ULK1
ENSG00000177182	157807	CLVS1
ENSG00000177291	219770	GJD4
ENSG00000177575	9332	CD163
ENSG00000177879	1176	AP3S1
ENSG00000177885	2885	GRB2
ENSG00000177938	93661	CAPZA3
ENSG00000177951	51272	BET1L
ENSG00000178038	259173	ALS2CL

ENSG00000178462	79861	TUBAL3
ENSG00000178950	2580	GAK
ENSG00000178996	112574	SNX18
ENSG00000179218	811	CALR
ENSG00000179331	54734	RAB39A
ENSG00000179562	79571	GCC1
ENSG00000179954	284297	SSC5D
ENSG00000180104	11336	EXOC3
ENSG00000180398	90411	MCFD2
ENSG00000181029	126003	TRAPPC5
ENSG00000181072	1129	CHRM2
ENSG00000181704	286451	YIPF6
ENSG00000181789	22820	COPG1
ENSG00000181856	6517	SLC2A4
ENSG00000181929	5571	PRKAG1
ENSG00000182287	8905	AP1S2
ENSG00000182400	122553	TRAPPC6B
ENSG00000182473	23265	EXOC7
ENSG00000182585	255324	EPGN
ENSG00000182827	64746	ACBD3
ENSG00000182963	10052	GJC1
ENSG00000183011	84316	NAA38
ENSG00000183020	161	AP2A2
ENSG00000183785	51807	TUBA8
ENSG00000184009	71	ACTG1
ENSG00000184014	23258	DENND5A
ENSG00000184381	8398	PLA2G6
ENSG00000184432	9276	COPB2
ENSG00000184840	54732	TMED9
ENSG00000185010	2157	F8
ENSG00000185236	9230	RAB11B
ENSG00000185359	9146	HGS
ENSG00000185963	23299	BICD2
ENSG00000186111	23396	PIP5K1C
ENSG00000186185	146909	KIF18B
ENSG00000186222	55330	BLOC1S4
ENSG00000187239	23048	FNBP1
ENSG00000187498	1282	COL4A1
ENSG00000187513	2701	GJA4
ENSG00000187714	6572	SLC18A3
ENSG00000187994	126432	RINL
ENSG00000188021	29978	UBQLN2
ENSG00000188229	10383	TUBB4B
ENSG00000188419	1121	CHM

ENSG00000188536	3040	HBA2
ENSG00000188559	57186	RALGAPA2
ENSG00000188910	2707	GJB3
ENSG00000189114	388552	BLOC1S3
ENSG00000189280	2709	GJB5
ENSG00000189433	127534	GJB4
ENSG00000196169	124602	KIF19
ENSG00000196352	1604	CD55
ENSG00000196459	6399	TRAPPC2
ENSG00000196547	4122	MAN2A2
ENSG00000196586	4646	MYO6
ENSG00000196655	51399	TRAPPC4
ENSG00000196814	89853	MVB12B
ENSG00000196961	160	AP2A1
ENSG00000197081	3482	IGF2R
ENSG00000197102	1778	DYNC1H1
ENSG00000197122	6714	SRC
ENSG00000197249	5265	SERPINA1
ENSG00000197535	4644	MYO5A
ENSG00000197694	6709	SPTAN1
ENSG00000197879	4641	MYO1C
ENSG00000197892	23303	KIF13B
ENSG00000197959	26052	DNM3
ENSG00000198033	7278	TUBA3C
ENSG00000198162	10905	MAN1A2
ENSG00000198399	50618	ITSN2
ENSG00000198612	10920	COPS8
ENSG00000198668	801	CALM1
ENSG00000198734	2153	F5
ENSG00000198835	57165	GJC2
ENSG00000198837	9909	DENND4B
ENSG00000198898	830	CAPZA2
ENSG00000203668	1122	CHML
ENSG00000203879	2664	GDI1
ENSG00000204070	90196	SYS1
ENSG00000204843	1639	DCTN1
ENSG00000205302	6643	SNX2
ENSG00000205593	414918	DENND6B
ENSG00000205726	6453	ITSN1
ENSG00000205744	79958	DENND1C
ENSG00000206172	3039	HBA1
ENSG00000206418	201475	RAB12
ENSG00000206560	23243	ANKRD28
ENSG00000211598	28908	IGKV4-1

ENSG00000211599	28907	IGKV5-2
ENSG00000211625	28874	IGKV3D-20
ENSG00000211640	28778	IGLV6-57
ENSG00000211644	28820	IGLV1-51
ENSG00000211648	28822	IGLV1-47
ENSG00000211651	28823	IGLV1-44
ENSG00000211652	28776	IGLV7-43
ENSG00000211653	28825	IGLV1-40
ENSG00000211658	28791	IGLV3-27
ENSG00000211659	28793	IGLV3-25
ENSG00000211660	28813	IGLV2-23
ENSG00000211662	28796	IGLV3-21
ENSG00000211663	28797	IGLV3-19
ENSG00000211666	28815	IGLV2-14
ENSG00000211668	28816	IGLV2-11
ENSG00000211673	28809	IGLV3-1
ENSG00000211677	3538	IGLC2
ENSG00000211679	3539	IGLC3
ENSG00000211890	3494	IGHA2
ENSG00000211895	3493	IGHA1
ENSG00000211934	28474	IGHV1-2
ENSG00000211937	28457	IGHV2-5
ENSG00000211938	28452	IGHV3-7
ENSG00000211941	28450	IGHV3-11
ENSG00000211942	28449	IGHV3-13
ENSG00000211949	28442	IGHV3-23
ENSG00000211955	28434	IGHV3-33
ENSG00000211956	28395	IGHV4-34
ENSG00000211959	28394	IGHV4-39
ENSG00000211962	28465	IGHV1-46
ENSG00000211964	28424	IGHV3-48
ENSG00000211967	28420	IGHV3-53
ENSG00000211973	28461	IGHV1-69
ENSG00000213047	163486	DENND1B
ENSG00000213380	84342	COG8
ENSG00000213983	8906	AP1G2
ENSG00000220205	6844	VAMP2
ENSG00000221838	9179	AP4M1
ENSG00000221983	7311	UBA52
ENSG00000223501	6293	VPS52
ENSG00000224373	28392	IGHV4-59
ENSG00000226650	285643	KIF4B
ENSG00000237649	3833	KIFC1
ENSG00000239264	81567	TXNDC5

ENSG00000239571	28881	IGKV2D-30
ENSG00000239951	28912	IGKV3-20
ENSG00000239975	28896	IGKV1D-33
ENSG00000240382	28937	IGKV1-17
ENSG00000240864	28938	IGKV1-16
ENSG00000241244	28901	IGKV1D-16
ENSG00000241351	28914	IGKV3-11
ENSG00000241553	10093	ARPC4
ENSG00000241685	10552	ARPC1A
ENSG00000242076	28933	IGKV1-33
ENSG00000242247	26286	ARFGAP3
ENSG00000242371	28930	IGKV1-39
ENSG00000242534	28883	IGKV2D-28
ENSG00000243238	28919	IGKV2-30
ENSG00000243244	11037	STON1
ENSG00000243290	28940	IGKV1-12
ENSG00000243466	28299	IGKV1-5
ENSG00000243910	80086	TUBA4B
ENSG00000244116	28921	IGKV2-28
ENSG00000244437	28913	IGKV3-15
ENSG00000244734	3043	HBB
ENSG00000251039	28878	IGKV2D-40
ENSG00000251546	28893	IGKV1D-39
ENSG00000254505	29082	CHMP4A
ENSG00000254647	3630	INS
ENSG00000257017	3240	HP
ENSG00000257923	1523	CUX1
ENSG00000258947	10381	TUBB3
ENSG00000261456	347688	TUBB8
ENSG00000261701	3250	HPR
ENSG00000264364	140735	DYNLL2
ENSG00000265107	2702	GJA5
ENSG00000265808	9554	SEC22B
ENSG00000270550	28439	IGHV3-30
ENSG00000274576	28454	IGHV2-70
ENSG00000276600	338382	RAB7B
ENSG00000278196	28817	IGLV2-8
ENSG00000278857	28903	IGKV1D-12

Table S3. Candidate prognostic genes screened from the VMTRGs genes

Gene Symbol	Coef	p.Value	HR (95% CI for HR)
ACTG1	0.397287	0.00047	1.5 (1.2-1.9)
ACTR10	0.515261	0.00073	1.7 (1.2-2.3)
ACTR1A	0.472561	0.0044	1.6 (1.2-2.2)

ACTR3	0.441982	0.00055	1.6 (1.2-2)
AGFG1	0.55386	1.10E-05	1.7 (1.4-2.2)
ANKRD27	0.319178	0.0016	1.4 (1.1-1.7)
AP1B1	0.510443	0.00076	1.7 (1.2-2.2)
AP1S2	0.346431	0.0024	1.4 (1.1-1.8)
AP1S3	0.61735	5.00E-06	1.9 (1.4-2.4)
AP2A2	0.618087	0.00058	1.9 (1.3-2.6)
AP2B1	0.429966	3.00E-04	1.5 (1.2-1.9)
AP2M1	0.426274	0.00063	1.5 (1.2-2)
AP2S1	0.370975	0.0046	1.4 (1.1-1.9)
AP3B1	0.387098	0.0041	1.5 (1.1-1.9)
AP3S1	0.417416	0.0032	1.5 (1.2-2)
AP4B1	0.77895	3.10E-05	2.2 (1.5-3.1)
AP4M1	0.491678	6.80E-05	1.6 (1.3-2.1)
ARCN1	0.380895	0.0038	1.5 (1.1-1.9)
ARF4	0.444684	0.00042	1.6 (1.2-2)
ARFGAP1	0.486117	0.00047	1.6 (1.2-2.1)
ARFIP2	0.573861	0.00013	1.8 (1.3-2.4)
ARFRP1	0.422664	0.0041	1.5 (1.1-2)
ARPC1A	0.306224	0.003	1.4 (1.1-1.7)
ARPC2	0.541315	7.90E-05	1.7 (1.3-2.2)
ARPC3	0.422697	0.0017	1.5 (1.2-2)
ARPC4	0.470899	6.90E-05	1.6 (1.3-2)
ARPC5	0.467179	0.00065	1.6 (1.2-2.1)
BET1L	0.516552	0.0032	1.7 (1.2-2.4)
BICD1	0.622888	1.30E-05	1.9 (1.4-2.5)
BLOC1S4	0.527701	7.80E-05	1.7 (1.3-2.2)
BNIP1	0.43476	0.0047	1.5 (1.1-2.1)
CALM1	0.460739	0.0049	1.6 (1.1-2.2)
CAPZA1	0.486058	0.00017	1.6 (1.3-2.1)
CAPZA2	0.370039	0.002	1.4 (1.1-1.8)
CAPZB	0.544939	0.00048	1.7 (1.3-2.3)
CBL	0.340416	0.0036	1.4 (1.1-1.8)
CCZ1B	0.513703	0.0033	1.7 (1.2-2.4)
CENPE	0.51879	1.70E-07	1.7 (1.4-2)
CHML	0.30183	0.00014	1.4 (1.2-1.6)
CHMP3	0.318562	0.0026	1.4 (1.1-1.7)
CHMP4B	0.475878	0.00024	1.6 (1.2-2.1)
CLTA	0.585136	9.10E-06	1.8 (1.4-2.3)
CLTC	0.45255	0.00041	1.6 (1.2-2)
COG1	0.390996	0.0043	1.5 (1.1-1.9)
COG4	0.594731	0.00011	1.8 (1.3-2.5)
COLEC12	0.221804	0.0024	1.2 (1.1-1.4)
COPA	0.355636	0.002	1.4 (1.1-1.8)

COPB1	0.433477	0.00075	1.5 (1.2-2)
COPB2	0.47644	0.001	1.6 (1.2-2.1)
COPG1	0.606917	0.00017	1.8 (1.3-2.5)
COPS6	0.478806	0.00077	1.6 (1.2-2.1)
COPS7B	0.600448	3.30E-05	1.8 (1.4-2.4)
COPS8	0.698638	4.00E-05	2 (1.4-2.8)
COPZ1	0.548285	0.00086	1.7 (1.3-2.4)
CSNK1D	0.589937	6.70E-05	1.8 (1.3-2.4)
CTSC	0.271986	0.00022	1.3 (1.1-1.5)
CYTH2	0.399124	0.0016	1.5 (1.2-1.9)
DAB2	0.28951	3.10E-05	1.3 (1.2-1.5)
DCTN2	0.585509	2.00E-05	1.8 (1.4-2.3)
DCTN5	0.527186	0.00012	1.7 (1.3-2.2)
DCTN6	0.39611	0.0048	1.5 (1.1-2)
DENND4B	0.353829	0.0029	1.4 (1.1-1.8)
DENND5A	0.435571	0.00034	1.5 (1.2-2)
DENND6A	0.554289	0.00025	1.7 (1.3-2.3)
DNAJC6	0.302172	0.00038	1.4 (1.1-1.6)
DNM1	0.223794	0.0031	1.3 (1.1-1.5)
DTNBP1	0.337232	0.0014	1.4 (1.1-1.7)
DVL2	0.359583	0.0017	1.4 (1.1-1.8)
DYNC1H1	0.460096	9.50E-05	1.6 (1.3-2)
DYNC1LI1	0.855738	7.40E-09	2.4 (1.8-3.1)
DYNLL1	0.674319	1.10E-05	2 (1.5-2.7)
EGF	0.296323	0.0036	1.3 (1.1-1.6)
EXOC1	0.468312	0.0014	1.6 (1.2-2.1)
EXOC3	0.504562	0.00087	1.7 (1.2-2.2)
GDI1	0.397972	0.0028	1.5 (1.1-1.9)
GDI2	0.792753	3.10E-06	2.2 (1.6-3.1)
GGA1	0.413544	0.001	1.5 (1.2-1.9)
GGA3	0.40437	0.0018	1.5 (1.2-1.9)
GJD4	3.642924	0.0029	38 (3.5-420)
GNS	0.321449	0.004	1.4 (1.1-1.7)
GOSR2	0.557101	0.00046	1.7 (1.3-2.4)
GPS1	0.453365	0.0026	1.6 (1.2-2.1)
GRB2	0.623138	3.70E-05	1.9 (1.4-2.5)
HPX	-0.12531	0.00064	0.88 (0.82-0.95)
HSP90AA1	0.452416	0.00016	1.6 (1.2-2)
HSPA8	0.426492	0.00018	1.5 (1.2-1.9)
HSPH1	0.383012	0.00062	1.5 (1.2-1.8)
KDELR1	0.558231	0.00011	1.7 (1.3-2.3)
KDELR3	0.182876	0.0028	1.2 (1.1-1.4)
KIF11	0.343549	1.00E-05	1.4 (1.2-1.6)
KIF15	0.380066	5.00E-06	1.5 (1.2-1.7)

KIF18A	0.485646	5.20E-08	1.6 (1.4-1.9)
KIF18B	0.314988	5.70E-06	1.4 (1.2-1.6)
KIF20A	0.381697	7.80E-08	1.5 (1.3-1.7)
KIF20B	0.326534	0.0025	1.4 (1.1-1.7)
KIF22	0.362203	0.00071	1.4 (1.2-1.8)
KIF23	0.35285	5.30E-06	1.4 (1.2-1.7)
KIF2A	0.599022	9.90E-06	1.8 (1.4-2.4)
KIF2C	0.376292	1.70E-08	1.5 (1.3-1.7)
KIF3A	0.541589	0.00025	1.7 (1.3-2.3)
KIF3C	0.277125	0.00035	1.3 (1.1-1.5)
KIF4A	0.296236	4.60E-06	1.3 (1.2-1.5)
KIF4B	7.990968	0.00032	3000 (38-230000)
KIFAP3	0.371499	0.0018	1.4 (1.1-1.8)
KIFC1	0.274106	1.10E-05	1.3 (1.2-1.5)
KLC2	0.356205	0.00089	1.4 (1.2-1.8)
LMAN2L	0.450777	0.0028	1.6 (1.2-2.1)
MADD	0.407724	0.0037	1.5 (1.1-2)
NBAS	0.496384	0.001	1.6 (1.2-2.2)
NECAP2	0.472452	0.0023	1.6 (1.2-2.2)
OPTN	0.449047	0.00066	1.6 (1.2-2)
PAFAH1B2	0.555193	0.00025	1.7 (1.3-2.3)
PAFAH1B3	0.196674	0.001	1.2 (1.1-1.4)
PPP6R1	0.32095	0.0042	1.4 (1.1-1.7)
PRKAG1	0.718555	8.30E-05	2.1 (1.4-2.9)
RAB10	0.491287	1.60E-05	1.6 (1.3-2)
RAB13	0.342598	0.0022	1.4 (1.1-1.8)
RAB32	0.455664	9.60E-06	1.6 (1.3-1.9)
RAB35	0.491427	5.00E-04	1.6 (1.2-2.2)
RAB38	0.279399	0.0011	1.3 (1.1-1.6)
RAB3IL1	0.279851	9.90E-05	1.3 (1.1-1.5)
RAB5C	0.567143	0.00035	1.8 (1.3-2.4)
RAB6B	0.335695	0.00073	1.4 (1.2-1.7)
RAB7A	0.744742	2.40E-05	2.1 (1.5-3)
RAC1	0.574148	7.80E-06	1.8 (1.4-2.3)
RACGAP1	0.356214	8.50E-06	1.4 (1.2-1.7)
RALA	0.585587	5.00E-05	1.8 (1.4-2.4)
SEC13	0.573605	0.00018	1.8 (1.3-2.4)
SEC22A	0.484962	0.0037	1.6 (1.2-2.3)
SEC22C	0.567377	0.00049	1.8 (1.3-2.4)
SFN	0.132575	3.00E-04	1.1 (1.1-1.2)
SH3GL1	0.443748	0.0011	1.6 (1.2-2)
SNAPIN	0.429884	0.0024	1.5 (1.2-2)
SNX2	0.486949	0.0022	1.6 (1.2-2.2)
SNX5	0.661729	1.70E-05	1.9 (1.4-2.6)

STAM	0.586448	1.10E-05	1.8 (1.4-2.3)
STX6	0.473052	9.00E-05	1.6 (1.3-2)
STXBP3	0.357734	0.0026	1.4 (1.1-1.8)
SURF4	0.518144	0.0023	1.7 (1.2-2.3)
SYS1	0.677395	5.10E-05	2 (1.4-2.7)
TBC1D10B	0.501682	0.00015	1.7 (1.3-2.1)
TBC1D13	0.358272	0.0031	1.4 (1.1-1.8)
TBC1D16	0.301225	0.0046	1.4 (1.1-1.7)
TBC1D20	0.444758	0.0046	1.6 (1.1-2.1)
TBC1D7	0.464827	0.003	1.6 (1.2-2.2)
TFG	0.554511	0.0011	1.7 (1.2-2.4)
TFRC	0.260139	0.0029	1.3 (1.1-1.5)
TMEM115	0.766464	1.50E-05	2.2 (1.5-3)
TRAPPC3	0.819643	3.80E-06	2.3 (1.6-3.2)
TRAPPC4	0.601709	3.20E-06	1.8 (1.4-2.4)
TSG101	0.556709	0.00096	1.7 (1.3-2.4)
TUBA1B	0.249059	0.0018	1.3 (1.1-1.5)
TUBA1C	0.370792	2.20E-05	1.4 (1.2-1.7)
TUBA4A	0.28768	0.00031	1.3 (1.1-1.6)
TUBA8	1.49578	0.003	4.5 (1.7-12)
TUBB4B	0.393812	0.0013	1.5 (1.2-1.9)
VPS25	0.469867	0.0018	1.6 (1.2-2.1)
VPS37C	0.528219	8.30E-05	1.7 (1.3-2.2)
VPS45	0.573847	3.80E-05	1.8 (1.4-2.3)
VTA1	0.479261	0.00076	1.6 (1.2-2.1)
YKT6	0.488606	3.70E-05	1.6 (1.3-2.1)
YWHAB	0.725014	1.40E-06	2.1 (1.5-2.8)
YWHAH	0.479244	6.70E-05	1.6 (1.3-2)
YWHAQ	0.426533	8.20E-05	1.5 (1.2-1.9)
YWHAZ	0.404465	5.80E-05	1.5 (1.2-1.8)
ZW10	0.45757	0.0019	1.6 (1.2-2.1)

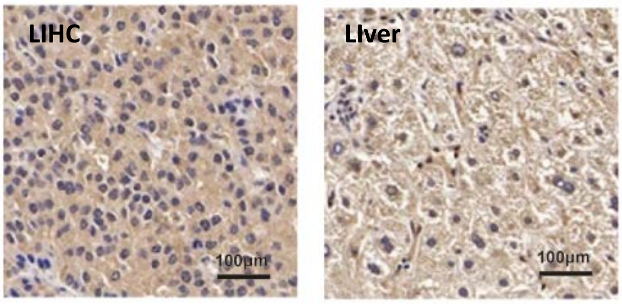

Figure S1. IHC staining of KIF2C in LIHC and normal liver tissue. The IHC staining images are from a reference (DOI: 10.1007/s00432-023-05079-1).

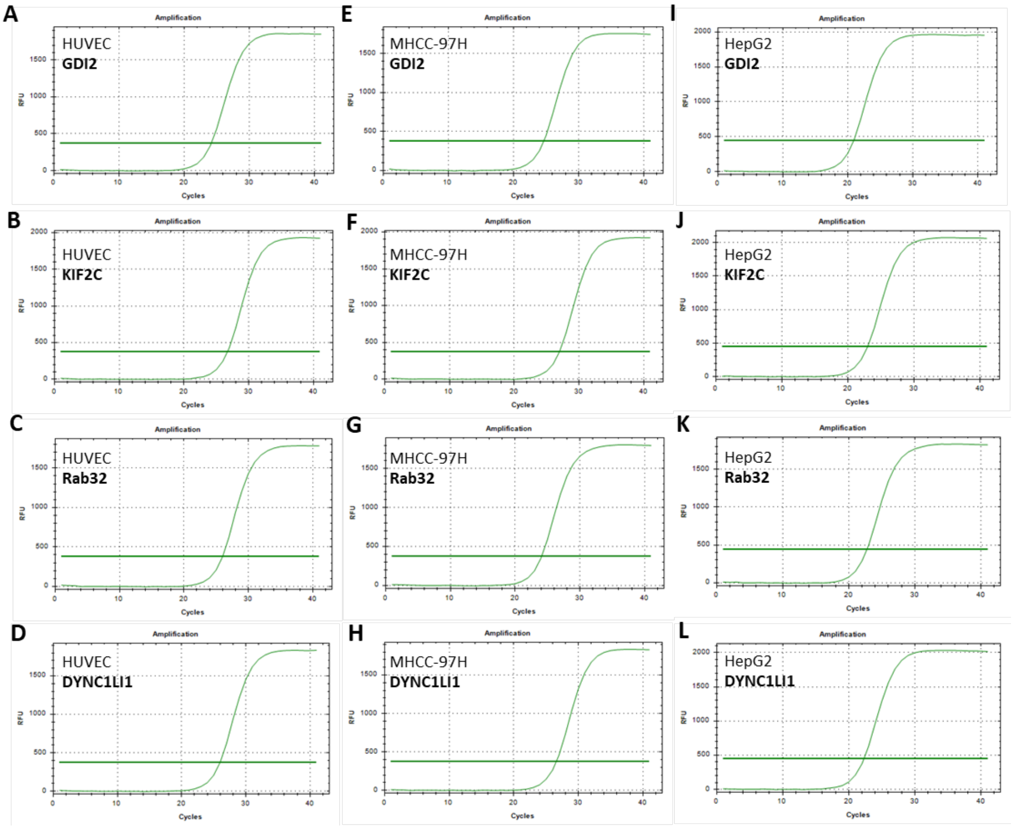

Figure S2. Amplification curve plots of DYNC1LI1, GDI2, KIF2C, and RAB32 genes in Q-PCR.

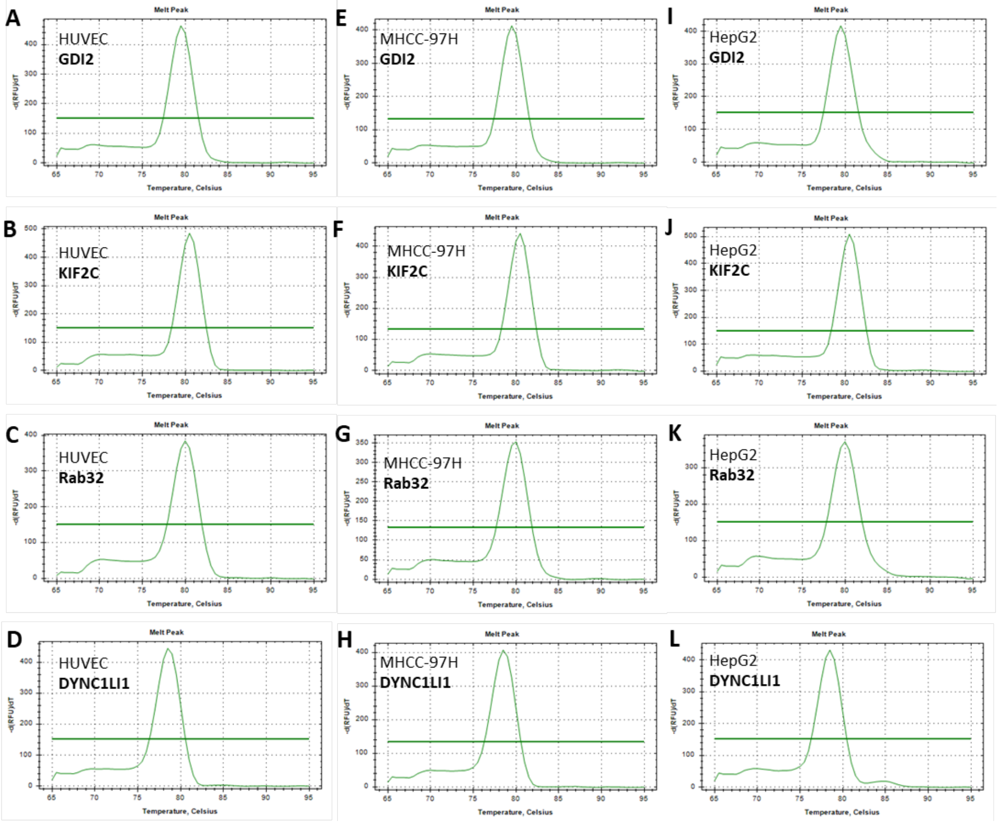

Figure S3. Melt curve plots of DYNC1LI1, GDI2, KIF2C, and RAB32 genes in Q-PCR.
